# Differential inputs to striatal cholinergic and parvalbumin interneurons imply functional distinctions

**DOI:** 10.7554/eLife.35657

**Published:** 2018-05-01

**Authors:** Jason R Klug, Max D Engelhardt, Cara N Cadman, Hao Li, Jared B Smith, Sarah Ayala, Elora W Williams, Hilary Hoffman, Xin Jin

**Affiliations:** 1 Molecular Neurobiology LaboratoryThe Salk Institute for Biological StudiesLa JollaUnited States; National Institute on Drug Abuse, National Institutes of HealthUnited States

**Keywords:** striatum, optogenetics, interneuron, rabies, ChAT, parvalbumin, Mouse

## Abstract

Striatal cholinergic (ChAT) and parvalbumin (PV) interneurons exert powerful influences on striatal function in health and disease, yet little is known about the organization of their inputs. Here using rabies tracing, electrophysiology and genetic tools, we compare the whole-brain inputs to these two types of striatal interneurons and dissect their functional connectivity in mice. ChAT interneurons receive a substantial cortical input from associative regions of cortex, such as the orbitofrontal cortex. Amongst subcortical inputs, a previously unknown inhibitory thalamic reticular nucleus input to striatal PV interneurons is identified. Additionally, the external segment of the globus pallidus targets striatal ChAT interneurons, which is sufficient to inhibit tonic ChAT interneuron firing. Finally, we describe a novel excitatory pathway from the pedunculopontine nucleus that innervates ChAT interneurons. These results establish the brain-wide direct inputs of two major types of striatal interneurons and allude to distinct roles in regulating striatal activity and controlling behavior.

## Introduction

Successful behavior requires the proper control of actions based on the integration of a wide variety of information, including both environmental stimuli and internal state, being processed across numerous brain regions. The basal ganglia, particularly the striatum, its main input nucleus, is a major node in the integration of multiple cortical and subcortical inputs underlying action control and learning (Graybiel, 2000; Hikosaka et al., 1999; Jin and Costa, 2015; Yin and Knowlton, 2006). The striatum is primarily composed of spiny projection neurons (SPNs), which consist of ~95% of total striatal neuronal population (Gerfen et al., 1990). Yet, intermixed throughout the striatum are local interneurons, which exert powerful regulation on SPN activity (Gittis et al., 2010; Goldberg et al., 2012; Schulz and Reynolds, 2013; Silberberg and Bolam, 2015; Tepper et al., 2010). This interneuron population includes large, aspiny choline acetyltransferase (ChAT)-positive cholinergic interneurons as well as a multitude of GABAergic interneuron subtypes differentiated based on expression of parvalbumin (PV), somatostatin, calretinin and other neurochemical markers (Tepper et al., 2010). Numerous studies have suggested that striatal ChAT and PV interneurons exhibit different membrane properties, connectivity and effects on modulating SPN activity (Gittis et al., 2010; Lim et al., 2014; Silberberg and Bolam, 2015; Tepper et al., 2010). However, compared to the different subtypes of SPNs (Smith et al., 2016; Wall et al., 2013) or the interneurons in cerebral cortex or hippocampus (Freund and Buzsáki, 1996; Kepecs and Fishell, 2014; Wall et al., 2016), relatively little is known about the organization of inputs to striatal interneurons let alone their precise function.

While ChAT interneurons account for only 1–2% of the total striatal population, the striatum contains some of the highest levels of cholinergic markers in the brain. Dysfunction of striatal ChAT interneurons has been implicated in numerous psychiatric disorders including schizophrenia, depression, and other mood disorders (Scarr et al., 2013), yet we lack a complete understanding regarding the input architecture to ChAT interneurons and their role in modifying behavior. ChAT interneurons are tonically active and exhibit a pause and subsequent rebound firing in response to the presentation of a cue predictive of reward or aversion (Ding et al., 2010; English et al., 2012; Kimura et al., 1984; Schulz and Reynolds, 2013). This conditioned pause is thought to encode the salience of external stimuli supporting the association of cue with action or outcome (Aosaki et al., 1994; Kimura et al., 1984; Ravel et al., 1999). Recently, striatal ChAT interneurons have been linked with behavioral flexibility and monitoring environmental state (Aoki et al., 2015; Apicella, 2007; Bradfield et al., 2013; Brown et al., 2010; Okada et al., 2014; Prado et al., 2017; Stalnaker et al., 2016). A vast majority of excitatory inputs to ChAT interneurons are thought to originate from centromedian and parafascicular nuclei of the thalamus (Ding et al., 2010; Lapper and Bolam, 1992); however, cortical stimulation has been shown to increase ChAT interneuron firing as well (Ding et al., 2010), suggesting a wealth of underappreciated cortical inputs.

PV interneurons (also known as fast-spiking interneurons) constitute ~1% of the total striatal neuronal population and their dysfunction has been implicated in multiple movement disorders and neuropsychiatric disorders including Huntington’s disease, dystonia, obsessive-compulsive disorder (OCD) and Tourette’s syndrome (TS) (Burguière et al., 2015; Kalanithi et al., 2005). Recent studies have suggested that PV interneurons increased their activity during action sequences and sensory-based decision making; however, their exact function in controlling behavior remains to be clearly determined (Adler et al., 2013; Gage et al., 2010; Jin et al., 2014). These neurons are capable of firing at very high rates *in vitro* and *in vivo* (Gage et al., 2010; Jin et al., 2014) providing strong feedforward inhibition onto SPNs (Gittis et al., 2010; Koós and Tepper, 1999). Striatal PV interneurons are believed to receive inputs primarily from cortex and globus pallidus, with minimal thalamic innervation (Bevan et al., 1998; Mallet et al., 2012; Saunders et al., 2016). However, the inputs to striatal PV interneurons have not been exhaustively mapped, which might be crucial for understanding their function in controlling behavior.

Here using rabies-mediated monosynaptic retrograde tracing and electrophysiology with optogenetics, we compare and analyze the whole-brain direct inputs to striatal ChAT and PV interneurons. Overall, ChAT and PV interneurons receive a vast majority of their inputs from the cortex. ChAT interneurons were found to preferentially receive inputs from association areas of cortex and thalamus. Among a wealth of different input targets revealed in the tracing study, we focus on the functional validation of three novel or underappreciated inputs to ChAT and PV interneurons. Specifically, a previously unknown inhibitory input from the thalamic reticular nucleus targeting PV interneurons was identified and functionally characterized. Additionally, an inhibitory pathway from the external segment of the globus pallidus to ChAT interneurons is explored, which robustly generates a pause in ChAT interneuron activity. We also identify a direct excitatory input from the pedunculopontine nucleus that targets ChAT interneurons. These results provide cell type-specific anatomical and functional connectivity for two major types of striatal interneurons providing insight into their role in controlling behavior.

## Results

### Monosynaptic tracing reveals the inputs to striatal ChAT and PV interneurons

**Figure 1.**
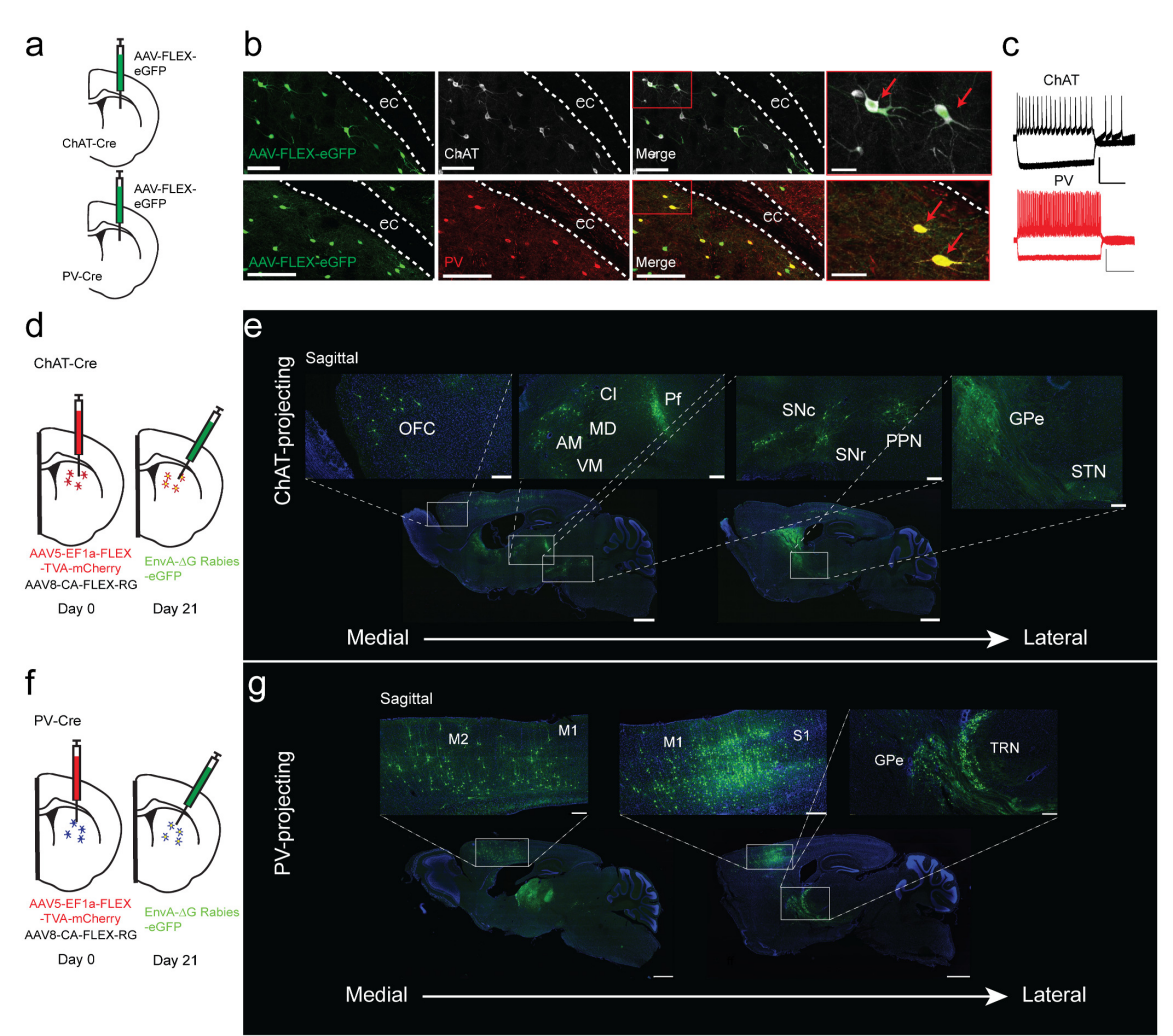
Monosynaptic tracing reveals the inputs to striatal ChAT and PV interneurons. (**a**) Schematic of Cre-dependent AAV-eGFP viral injection in ChAT-Cre or PV-Cre mice. (**b**) AAV-eGFP expression is highly colocalized with ChAT and PV immunostaining, respectively. Scale bars, 100 µm. inset (red box), scale bar 25 µm. Red arrows denote colocalization. ec, external capsule. (**c**) Representative traces of (top) ChAT and (bottom) PV interneuron to hyperpolarizing and depolarizing current injection (step −250 pA,+250 pA). Scale bars, 250 ms, 50 mV. (**d**) Schematic of Cre-dependent AAV helper viruses and modified rabies virus injections in ChAT-Cre mice. (**e**) Series of representative sagittal sections containing inputs to ChAT interneurons. (**f**) Schematic of Cre-dependent AAV helper viruses and modified rabies virus injections in PV-Cre mice. (**g**) Series of representative sagittal sections containing inputs to PV interneurons. Only the injection hemisphere is shown. Scale bars, 1 mm; inset scale bars; 500 µm. Brain regions are highlighted in white lettering. OFC, orbital frontal cortex; M1, primary motor cortex; M2, secondary motor cortex; S1, primary somatosensory cortex; GPe, globus pallidus external segment; Cl, central lateral thalamic nuclei; Pf, parafascicular thalamic nucleus; AM, anteromedial thalamic nucleus; MD, mediodorsal thalamic nucleus; VM, ventromedial thalamic nucleus; STN, subthalamic nucleus, SNc, substantia nigra pars compacta; SNr, substantia nigra pars reticulata; PPN, pedunculopontine nucleus; TRN, thalamic reticular nucleus. The following figure supplements are available for Figure 1: Figure 1—figure supplement 1 and Figure 1—figure supplement 2 for additional coronal images and starter cell quantification, respectively.

We used transgenic ChAT-Cre and PV-Cre mouse lines to target striatal ChAT and PV interneurons in the striatum. To validate Cre line specificity, a Cre-dependent AAV virus that expresses eGFP was injected into the dorsal striatum of either ChAT-Cre or PV-Cre mice (Figure 1a). The eGFP expression in striatum of ChAT-Cre and PV-Cre mice was highly specific as demonstrated by colocalization with immunohistochemical staining for choline acetyltransferase or parvalbumin (Figure 1a,b; Figure 1—figure supplement 1a–c, ChAT 95.9 ± 0.78%, PV 95.6 ± 0.79%). Additionally, *ex vivo* electrophysiological recordings of GFP-expressing ChAT or PV interneurons show typical electrophysiological properties (Figure 1c). ChAT interneurons are tonically active, have depolarized resting membrane potentials, and feature prominent hyperpolarization-activation cation currents (Figure 1c), while PV interneurons exhibit narrow action potentials and high firing rates (Gittis et al., 2010; Tepper et al., 2010). These results demonstrate that the ChAT-Cre and PV-Cre lines are highly selective in striatum and therefore appropriate for investigating the inputs to striatal ChAT and PV interneurons.

**Figure 1—figure supplement 1.**
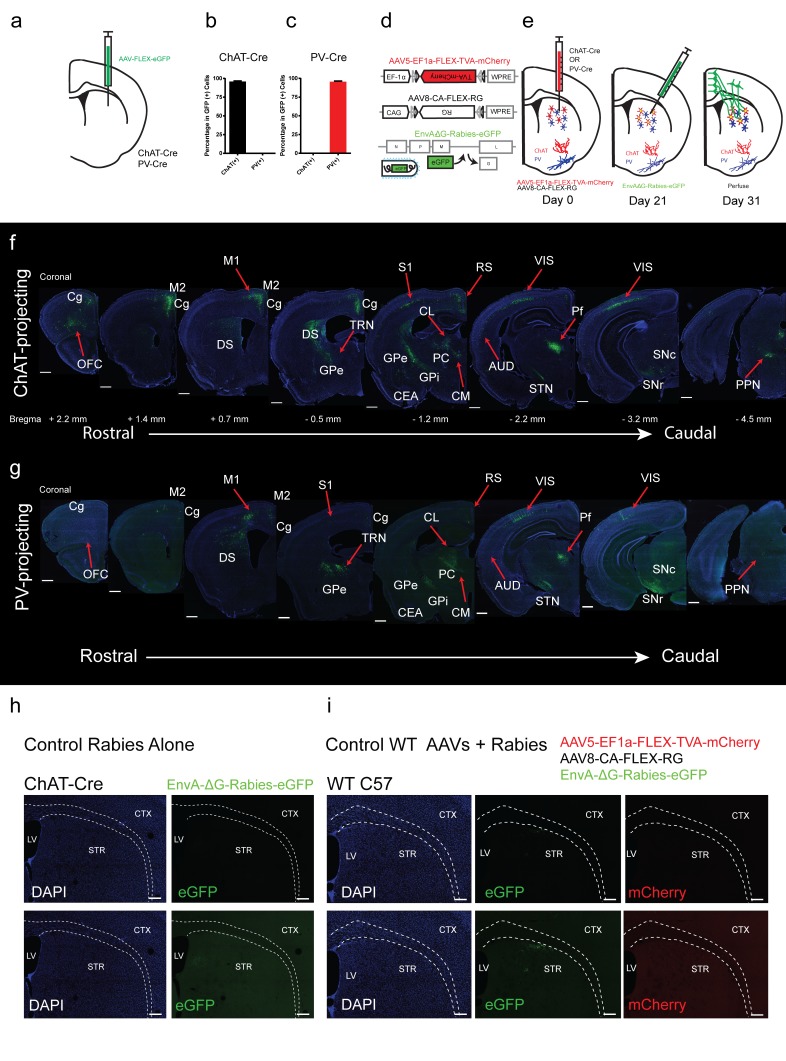
Overview of inputs to ChAT and PV interneurons. (**a**) Schematic of Cre-dependent AAV eGFP injection in striatum of ChAT-Cre or PV-Cre mouse. (**b**) Quantification of ChAT immunostaining and eGFP expression colocalization. (**c**) Quantification of PV immunostaining and eGFP expression colocalization. Mean ± SEM. (**d**) Schematic of recombinant Cre-dependent AAV helper viruses and pseudotyped modified rabies virus. (**e**) Experimental methodology and timeline of viral injections. (**f**) Series of representative coronal sections with modified rabies virus eGFP monosynaptic inputs to dorsal striatal ChAT interneurons. (**g**) Series of representative coronal sections with modified rabies virus eGFP monosynaptic inputs to dorsal striatal PV interneurons. Only the hemisphere ipsilateral to the injection site is shown. Scale bar, 500 µm. Areas of expression are highlighted in white lettering. Red arrows denote significant input differences in ChAT and PV neurons. OFC, orbital frontal cortex; Cg, cingulate cortex; M1, primary motor cortex; M2, secondary motor cortex; DS, dorsal striatum; S1, primary somatosensory cortex; S2, secondary somatosensory cortex; GPe, globus pallidus external segment; GPi, globus pallidus internal segment; CEA, central amygdala; CL, central lateral thalamic nuclei; PC, paracentral thalamic nuclei; CM, central medial thalamic nucleus; Pf, parafascicular thalamic nucleus; STN, subthalamic nucleus, RS, retrosplenial cortex; AUD, auditory cortex; VIS, visual cortex; SNc, substantia nigra pars compacta; SNr, substantia nigra pars reticulata; PPN, pedunculopontine nucleus; TRN, thalamic reticular nucleus. (**h**) (top) dG-Rabies injection alone in a ChAT-Cre mouse does not lead to eGFP expression. (top) same gain settings as f-g. (bottom) high gain settings. (**i**) Injection of helper viruses and dG-Rabies in a wildtype mouse does not yield significant expression of mCherry or eGFP. (top) same gain settings as f-g. (bottom) high gain settings. Scale bar, 200 µm. LV = lateral ventricle, STR = striatum, CTX = cortex.

**Figure 1—figure supplement 2.**
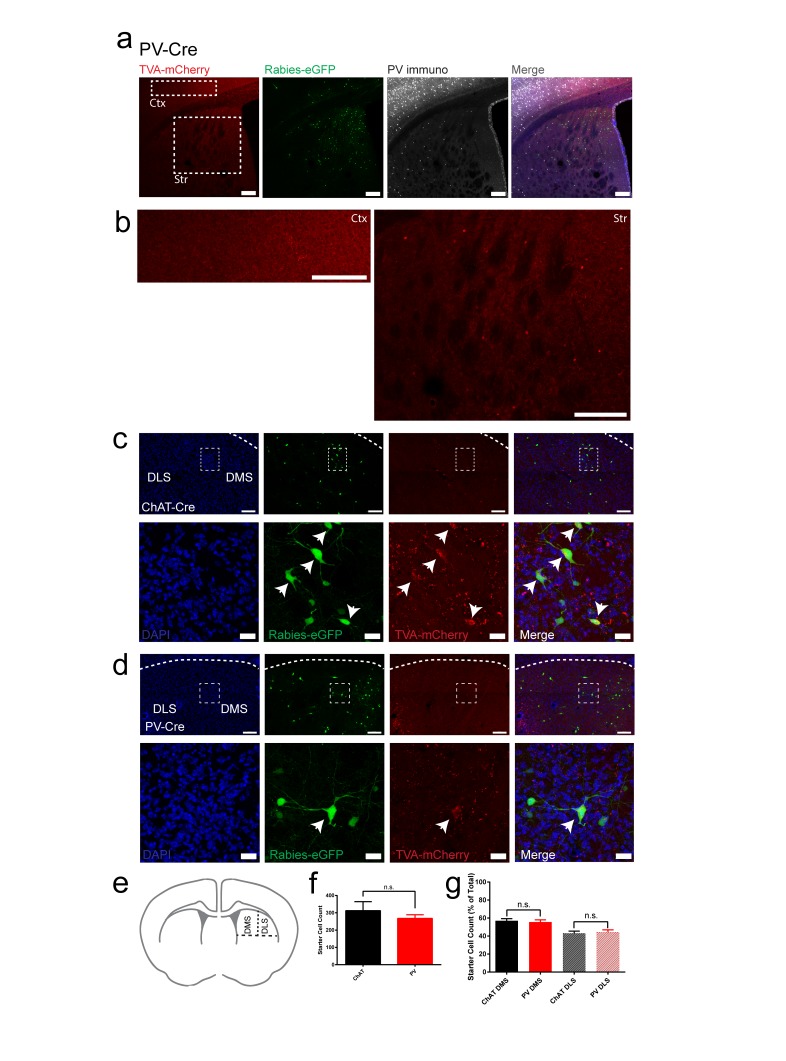
ChAT and PV starter cells are restricted to dorsal striatum, similar in total number and are distributed equally across dorsal medial and dorsal lateral striatal subdivisions. (**a**) Expression of TVA-mCherry positive starter cells, rabies-eGFP and immunostaining for PV in dorsal striatal injection site. Scale bar, 100 um. (**b**) Magnified inset of cortex (Ctx) and dorsal striatum (Str) from (**a**). Scale bar, 100 um. All PV-Cre rabies tracing brains were confirmed to not express TVA-mCherry positive starter cells in cortex. (**c**) Expression of Rabies-eGFP and TVA-mCherry in the dorsal striatum of a ChAT-Cre mouse near the injection site. (top) 20x image of dorsal striatum. Scale bar, 100 µm. White dotted rectangle inset (below) 40x image from above. Scale bar, 20 µm. Neurons with both eGFP and mCherry expression are considered starter cells (white arrowheads). (**d**) Expression of Rabies-eGFP and TVA-mCherry in the dorsal striatum of a PV-Cre mouse near the injection site. (top) 20x image of dorsal striatum. Scale bar, 100 µm. White dotted rectangle inset (below) 40x image from above. Scale bar, 20 µm. (**e**) Area of dorsal striatum where counts were collected and bisection of dorsal medial (DMS) and dorsal lateral (DLS) striatum. (**f**) Total number of starter cells in dorsal striatum from a sample of rostral to caudal slices. (**g**) Starter cell count for dorsal medial and dorsal lateral subdivisions presented as a percentage of total starter cells. n.s. = not significant.

**Figure 2.**
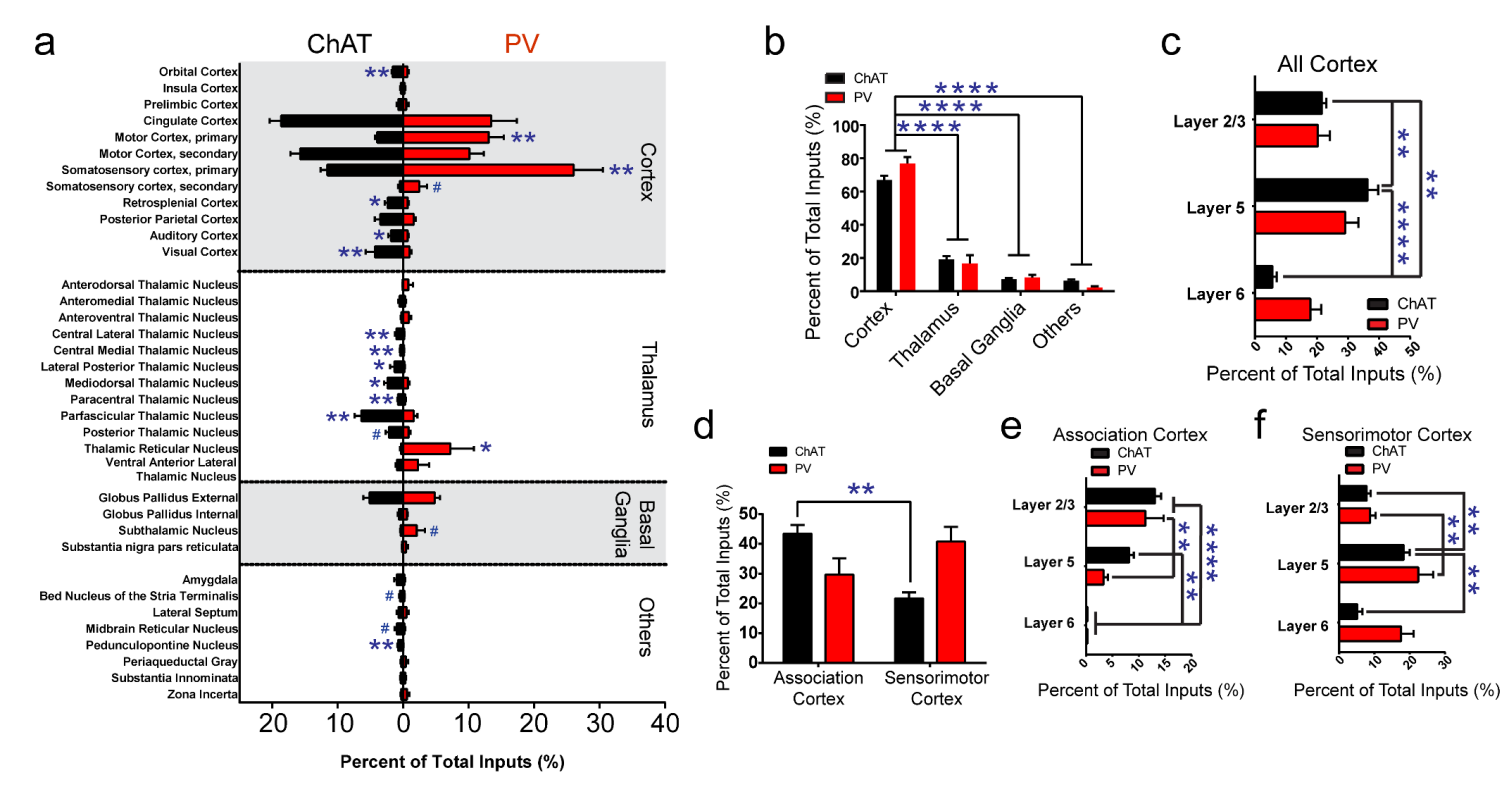
Summary of brain-wide direct inputs to striatal ChAT and PV interneurons. (**a**) Major input regions (>0.4% of total inputs) to ChAT (black bar) and PV (red bar) interneurons. A complete set of all inputs to ChAT and PV interneurons is shown in Figure 2—figure supplement 1. (**b**) ChAT and PV interneurons receive predominately cortical innervation (two-way ANOVA, F_(3,36)_ = 325.1, p<0.0001). (**c**) Cortical laminar distribution of all inputs to ChAT and PV interneurons. ChAT interneurons receive a significant bulk of inputs from layer five compared to other cortical layers (two-way ANOVA, Sidak’s, 5 vs. 6, p<0.0001; 2/3 vs. 6, p=0.0019; 2/3 vs. 5, p=0.0041). (**d**) Sorted association and sensorimotor cortex inputs to ChAT and PV interneurons (see Materials and methods). ChAT interneurons receive a greater percentage of associative versus sensorimotor inputs (two-way ANOVA, Sidak’s, p=0.0001). (**e**) Association cortex laminar distribution is biased to superficial layers. ChAT interneurons receive more associative layer 2/3 than layer six projections and more layer 5 than six projections (two-way ANOVA, Sidak’s, layer 2/3 vs. 6, p<0.0001, layer 5 vs. 6, p=0.0012). PV interneurons also receive more associative layer 2/3 than layer 5 and 6 projections (two-way ANOVA, Sidak’s, layer 2/3 vs. 6, p<0.0001, layer 2/3 vs 5, p=0.0029). (**f**) Sensorimotor cortex laminar distribution is biased to deep layers. ChAT interneurons receive more sensorimotor layer five projections than layer 2/3 or layer six projections (two-way ANOVA, Sidak’s, layer 5 vs. 6, p=0.0010, layer 2/3 vs. 5, p=0.0086). PV interneurons receive more sensorimotor layer 5 than layer 2/3 projections (two-way ANOVA, Sidak’s, layer 2/3 vs. 5, p=0.0018). All figures mean ± SEM. In blue, *p≤0.05, **p≤0.01, ****p≤0.0001, # p<0.08. The following figure supplements are available for Figure 2: Figure 2—figure supplements 1 and 2.

To determine the differences in inputs to striatal ChAT and PV interneurons, we performed Cre-dependent, modified rabies virus tracing (Guo et al., 2015; Wickersham et al., 2007) in the dorsal striatum of ChAT- or PV-Cre mice from unilateral viral injections into the same location (See Materials and methods, Figure 1d–g; Figure 1—figure supplement 1d–i; ChAT n = 6, PV n = 5). All starter cells were found to be restricted to the dorsal striatum without any cortical expression (Figure 1—figure supplement 2a,b). There was no difference in the total number or the striatal subregion distribution between ChAT and PV starter neurons (Figure 1—figure supplement 2c–g). In both Cre mice, eGFP-positive projection neurons were found throughout cortex, thalamus, basal ganglia and other subcortical regions (Figure 1e,g; Figure 1—figure supplement 2f,g). Labeling on the ipsilateral side throughout the brain was quantified relative to brain region boundaries (as defined by the Allen Institute Mouse Brain Reference Atlas). There was no significant difference in the total number of input neurons for ChAT and PV interneurons (ChAT 6580 ± 2657, PV 4456 ± 1540; two-tailed t-test, p=0.4773). A cutoff of greater than 0.4% was used to distinguish between major and minor inputs to these interneuron populations (Figure 2a for all major inputs, see a complete list in Figure 2—figure supplement 1 with a statistical comparison between ChAT and PV inputs).

**Figure 2—figure supplement 1.**
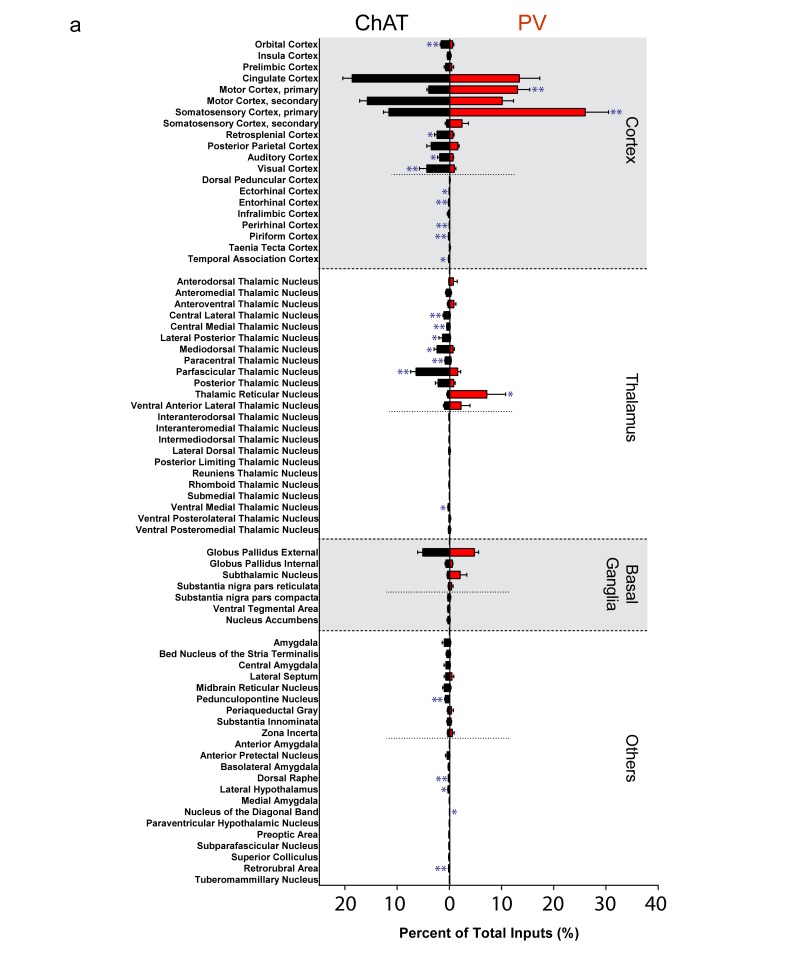
Complete list of all inputs to ChAT and PV interneurons. (**a**) All major (>0.4%) and minor (<0.4%) inputs to striatal ChAT (black) and PV (red) interneurons. Short dashed line represents division between major and minor inputs. Mean ± SEM. In blue, *p≤0.05; **p≤0.01.

**Figure 2—figure supplement 2.**
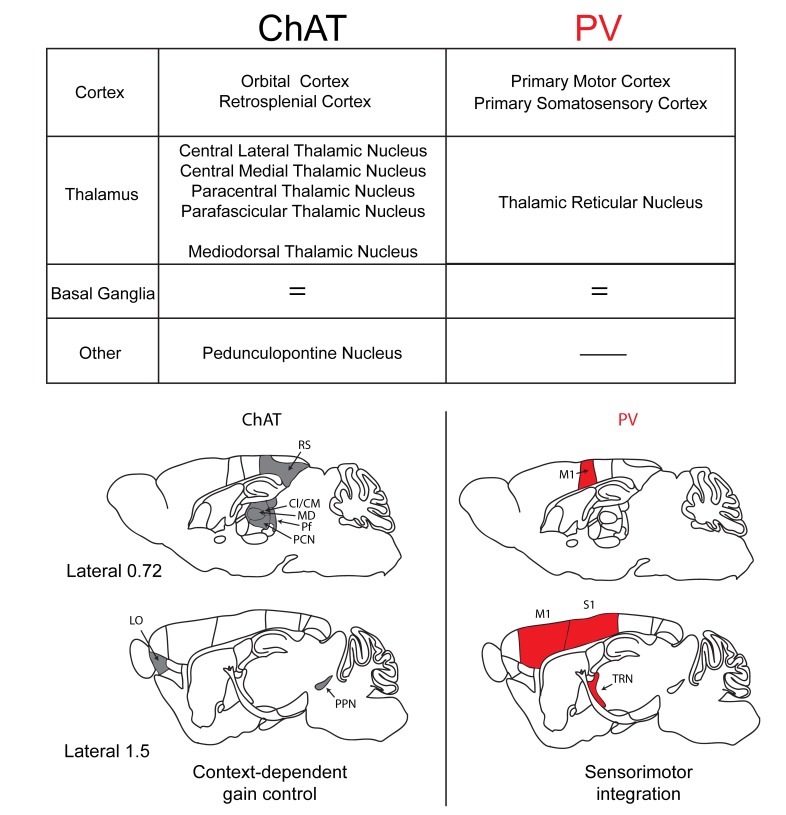
Summary of the inputs and proposed function of striatal ChAT and PV interneurons. Striatal ChAT interneurons receive preferential inputs from orbital cortex, retrosplenial cortex, intralaminar thalamic nuclei, mediodorsal thalamus and the pedunculopontine nucleus and might be associated with state-dependent gain control of actions. PV interneurons receive dominant inputs from primary motor cortex, primary somatosensory cortex and the thalamic reticular nucleus and might be associated with sensorimotor learning.

The tracing results revealed that both types of striatal interneurons received a vast majority of inputs from the cortex (Figure 2b, two-way ANOVA, *F*_(3, 36)_=325.1, p<0.0001), with the remaining inputs coming from various thalamic nuclei, other basal ganglia nuclei, and a variety of other subcortical regions. ChAT interneurons received substantial inputs from cingulate cortex and secondary motor cortex. PV interneurons received extensive inputs from similar cortical regions with the addition of a substantial primary motor and primary somatosensory cortical inputs (Figure 2a). We observed input neurons to ChAT or PV interneurons from layer 2/3, 5, and 6 of cortex (Figure 2c). ChAT interneurons received significantly more inputs from layer five compared to all other cortical layers and more layer 2/3 compared to layer six projections (Figure 2c; two-way ANOVA, Sidak’s, 5 vs. 6, p<0.0001; 2/3 vs. 6, p=0.0019; 2/3 vs. 5, p=0.0041).

When grouping the cortical inputs into association-like (e.g. orbital, insula, prelimbic, cingulate, M2, S2, RS, and PPC, see Materials and methods) and sensorimotor-like regions (e.g. M1, S1, AUD, VIS), ChAT interneurons received significantly more inputs from associative than sensorimotor cortex (Figure 2d, two-way ANOVA, Sidak’s multiple comparisons test, p=0.0001). Examination of the laminar distribution in associative versus sensorimotor cortical regions revealed a unique pattern. Inputs in associative cortex are superficial preferring (layer 2/3) while sensorimotor inputs are more prominent in deeper layers (layer 5/6) (Figure 2e,f) (Smith et al., 2016). Association cortex inputs to ChAT interneurons were layer 2/3 dominant with significantly more layer 2/3 inputs than layer six and more layer five inputs than layer 6 (Figure 2e; two-way ANOVA, Sidak’s, layer 2/3 vs. 6, p<0.0001, layer 5 vs. 6, p=0.0012). Similarly, PV interneurons association cortical inputs were more layer 2/3 preferring (Figure 2e; two-way ANOVA, Sidak’s, layer 2/3 vs. 6, p<0.0001, layer 2/3 vs 5, p=0.0029). Sensorimotor inputs to ChAT interneurons originated predominately from layer 5 (Figure 2f; two-way ANOVA, Sidak’s, layer 5 vs. 6, p=0.0010, layer 2/3 vs. 5, p=0.0086. PV interneurons also received significantly more sensorimotor layer 5 than layer 2/3 projections (Figure 2f; two-way ANOVA, Sidak’s, layer 2/3 vs. 5, p=0.0018). These results show that although both types of striatal interneurons receive more superficial association cortex inputs and more deep layer sensorimotor cortex inputs, ChAT interneurons receive more associative cortical inputs overall.

### Thalamic projections to striatal ChAT and PV interneurons

The previous literature on thalamostriatal projections suggests that intralaminar nuclei mainly target striatal ChAT interneurons or SPNs (Ding et al., 2010; Smith et al., 2004), however a growing body of work also suggest intralaminar thalamic nuclei also target PV interneurons (Arias-García et al., 2017; Assous et al., 2017; Sciamanna et al., 2015; Sidibé and Smith, 1999). While all four intralaminar nuclei had inputs targeting ChAT interneurons, our dG-rabies tracing revealed that PV interneurons also received significant projections from intralaminar thalamus (Figure 2a). Interestingly, ChAT interneurons have inputs from all intralaminar nuclei and associative thalamic nuclei like mediodorsal thalamus (Figure 2a). Together, these data suggest that like association cortex, associative thalamic nuclei and intralaminar nuclei also have a substantial proportion of inputs targeting striatal ChAT interneurons.

**Figure 3.**
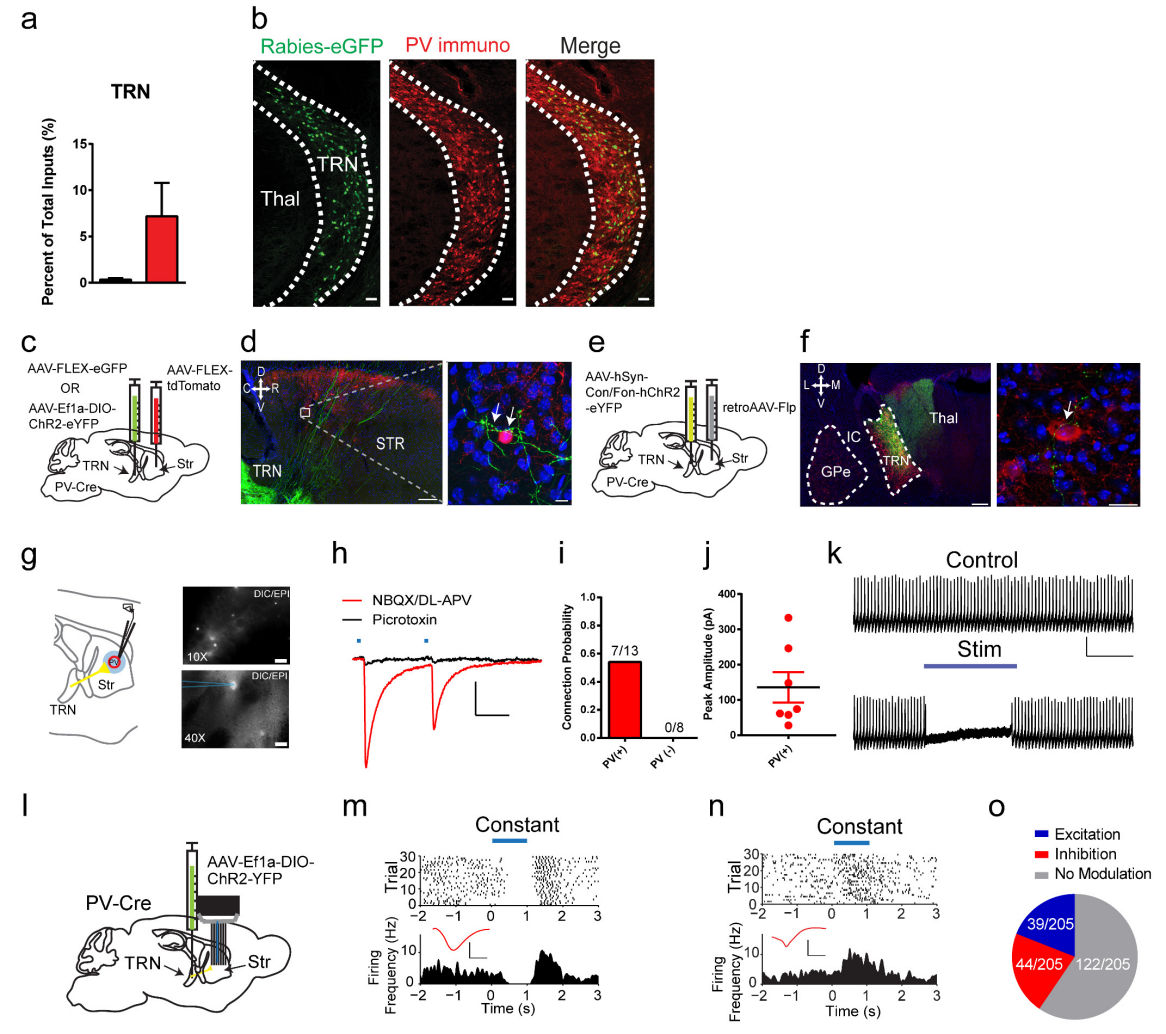
A projection from thalamic reticular nucleus to striatal PV interneurons. (**a**) PV interneurons receive a thalamic reticular nucleus (TRN) projection. (**b**) Rabies-eGFP positive neurons in TRN colocalize with PV immunostaining. Scale bar, 50 µm. Thal = thalamus. (**c**) Anterograde viral injection scheme to validate TRN to PV interneuron projection. (**d**) (left) Cre-dependent AAV eGFP is injected in TRN and Cre-dependent AAV tdTomato injected in the dorsal striatum of a PV-Cre mouse. Scale 200 µm. (right) 63x image of eGFP positive terminals on PV interneuron (red) somas and proximal dendrites (white arrows). Scale 10 µm. (**e**) A convergent intersectional viral approach to trace TRN projections to striatum. (**f**) (left) Expression of eYFP in anterior TRN cell bodies and axons seen projecting to dorsal thalamus. Note lack of expression in neighboring GPe. Scale bar, 200 µm. (right) Following PV immunostaining, eYFP fibers (yellow-green) from TRN were observed in close apposition to PV cell somas (red). Scale bar, 20 µm. (**g**) (left) Slice preparation to functionally validate TRN to PV interneuron projections. (right) Overlays of DIC and red channel epifluorescence at 10x (top) and 40x (bottom). Scale bars 200 µm, 20 µm. (**h**) Whole-cell recordings of paired light evoked IPSCs (50 ms ISI) following TRN terminal stimulation. IPSCs are blocked with GABA_A_R antagonist, picrotoxin (50 µM). Scale bars, 25 ms, 100 pA. PV (+) cells are held at −70 mV. (**i**) Striatal PV interneurons showed a fast latency, reliable light evoked IPSC, which is absent in the neighboring SPNs. (**j**) Individual IPSC current amplitudes of connected cells. (**k**) One second constant blue laser TRN axon stimulation is sufficient to suppress current injection-induced spiking in a PV interneuron. Scale bars, 500 ms, 25 mV. (**l**) Optogenetic stimulation of TRN terminals in the striatum was paired with extracellular neuronal recordings using a multi-electrode array. (**m–n**) Exemplar (top) spike raster and (bottom) firing frequency perievent time histogram (PETH) from a SPN exhibiting slow latency light-evoked inhibition (**m**), or exhibiting slow latency light-evoked excitation (**n**). Inset (red): Average single unit waveforms of a putative SPN. Scale bars 0.2 ms, 50µV. (**o**) Approximately 40% (83/205) of neurons show light-induced firing rate modulation. Half of the responsive SPN population (44/83) exhibited delayed inhibition (red), while others (39/83) exhibited slow latency excitation (blue). See Figure 3—figure supplement 3 for putative PV interneuron light-evoked responses. The following figure supplements are available for Figure 3: Figure 3—figure supplements 1–3.

Our dG-rabies tracing surprisingly revealed that PV interneurons receive a projection from the thalamic reticular nucleus (TRN) (Figure 2a, Figure 3a). The TRN is a thin shell of GABAergic neurons that was thought to project within thalamus regulating thalamocortical and corticothalamic communications (Halassa and Acsády, 2016). The rabies-eGFP signal was clearly located within the TRN as evidenced by colocalization with parvalbumin (PV) or somatostatin (SOM) immunostaining (Clemente-Perez et al., 2017) (Figure 3b, Figure 3—figure supplement 1e,f, PV: rabies-eGFP colocalization 33.44 ± 6.08%, SOM: rabies-eGFP colocalization 43.73 ± 3.88%), and was enriched in the most rostral pole of the TRN. Noticeably, the labeling of TRN was never observed in control experiments with either helper or rabies virus alone nor were TVA-mCherry starter cells observed in TRN (Figure 1—figure supplement 1h,i). In order to anatomically validate this projection from TRN to PV interneurons independently, three additional viral tracing experiments were performed. In the first approach, anterograde tracing from TRN in PV-Cre mice with a Cre-dependent AAV expressing eGFP (Figure 3c), showed that labeled TRN neurons sent projections coursing rostrally into the striatum and form close appositions to PV interneuron somas and proximal dendrites (Figure 3d). Secondly, using a convergent intersectional approach to trace TRN projections, a retrograde AAV virus (Tervo et al., 2016) carrying Flp-recombinase was injected into dorsal striatum of a PV-Cre mouse, while a dual necessity Cre-/Flp- recombinant INTRSECT AAV virus was injected in TRN (Figure 3e). Following transfection, eYFP-expressing cell bodies were seen in the anterior TRN, but not surrounding areas like GPe, with clear axonal projections observed in the dorsal thalamus (Figure 3f). Utilizing striatal PV immunostaining, eYFP fibers from TRN were observed to be in close apposition to PV cell somas in the striatum (Figure 3f). As an additional confirmation of TRN to striatal connectivity we injected Somatostatin-IRES-Cre mice with Cre-dependent AAV-FLEX-eGFP to label TRN neurons (Figure 3—figure supplement 1g–i). TRN expressed eGFP selectively and eGFP-positive fibers can be seen near PV interneurons in striatum. Thus, these three viral tracing experiments have independently verified the rabies tracing results and confirmed a previously unknown projection from TRN to striatal PV interneurons.

**Figure 3—figure supplement 1.**
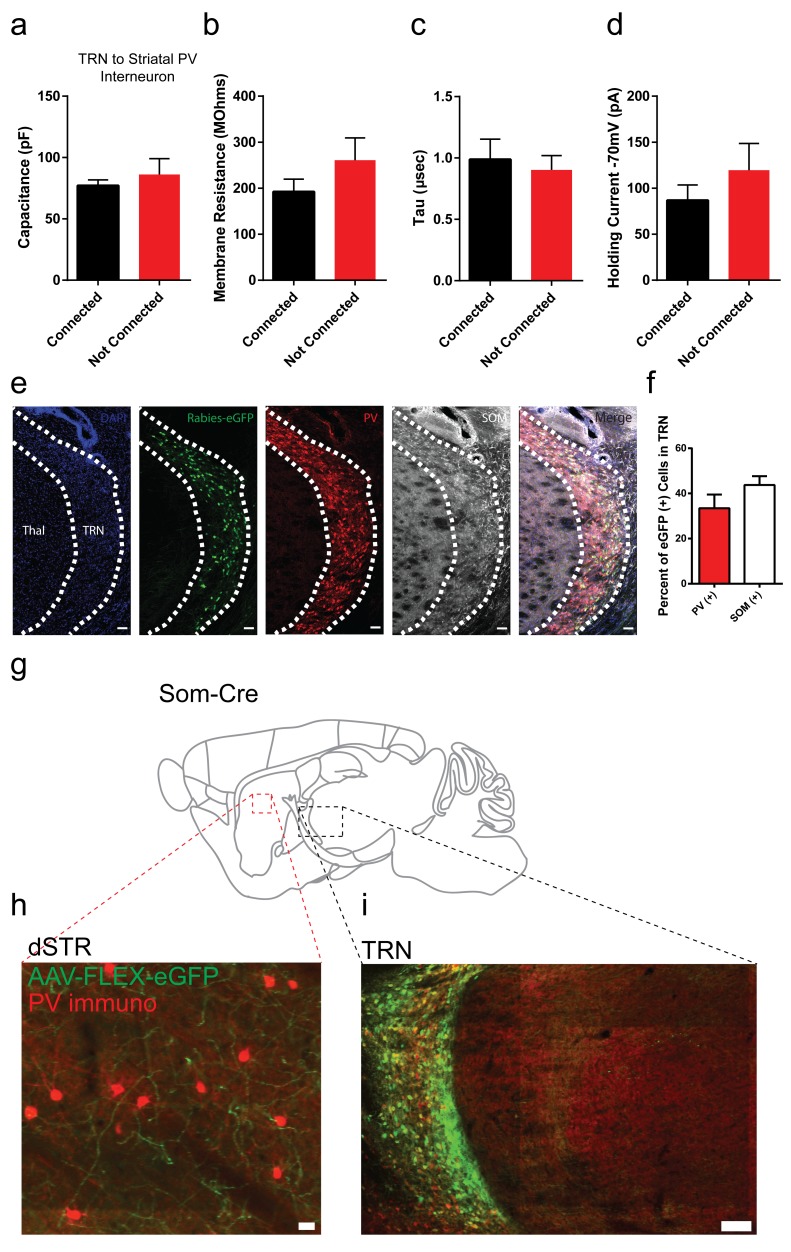
Membrane properties of striatal PV interneurons are similar between TRN connected or non-connected cells. Rabies positive eGFP cells in TRN colocalize with parvalbumin and somatostatin immunostaining. Somatostatin (Som) positive TRN neurons send projections to striatum. (**a–d**) Capacitance, membrane resistance, decay tau and holding current of TRN-connected or TRN non-connected PV striatal neurons is not significantly different (two-tailed t-test, p=0.5008, 0.2262, 0.6925, 0.3311). (**e**) Sagittal image of rabies-eGFP positive neurons in TRN with immunostaining for parvalbumin (red) and somatostatin (grey). Scale bar, 50 µm. Thal = thalamus. (**f**) Quantification of rabies-eGFP colocalization in TRN with parvalbumin (PV) and somatostatin (SOM) immunostaining. (**g**) Cartoon sagittal mouse brain slice from a Somatostatin-IRES-Cre mouse. (**h**) Image of dorsal striatum with Cre-dependent AAV-eGFP terminals from TRN (green) near immunopositive PV interneurons (red). Scale bar, 20 µm. (**i**) Image of TRN with Cre-dependent AAV-eGFP expression (green) and PV immunostaining (red). Scale bar, 100 µm.

**Figure 3—figure supplement 2.**
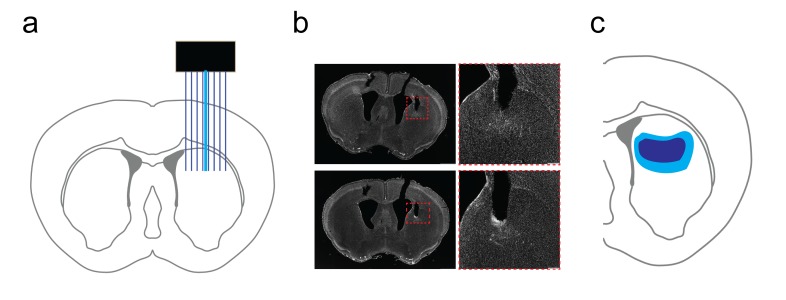
Verification of dorsal striatal in vivo recording array placement. (**a**) Cartoon schematic of multichannel array placement with optical fiber for light delivery in dorsal striatum. (**b**) Grayscale DAPI stained images of striatal slices (left) following *in vivo* recording with optogenetic stimulation. Scale bar, 1 mm. (Right) inset image of recording area in dashed red box on left. Scale bar, 200 µm. Note the track marking the dorsal boundary of the recording area. (**c**) Graphical representation of minimum (dark blue) and maximal (light blue) territories for *in vivo* array placement in dorsal striatum.

**Figure 3—figure supplement 3.**
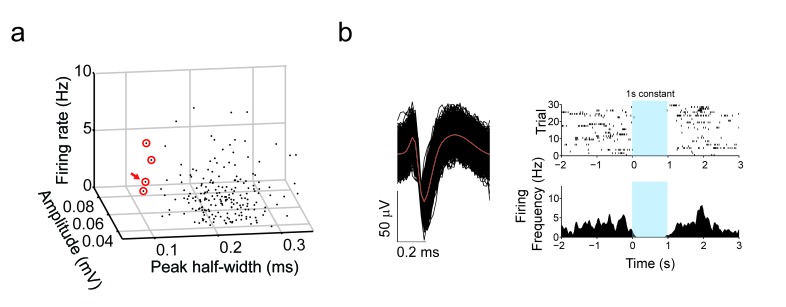
TRN terminal stimulation inhibits putative striatal fast-spiking (PV) interneurons. (**a**) 3D plot of all recorded units by peak half-width (ms), amplitude (mV) and firing rate (Hz). Putative PV interneurons sorted by narrow half widths (<0.12 ms). Red circles denotes putative fast spiking interneurons. Red arrow denotes putative fast spiking interneuron in shown in (**b**). (**b**) Example of (left) narrow spike waveform of putative fast-spiking (PV) interneurons from *in vivo* recordings in dorsal striatum of PV-Cre mice (n = 4). Red line denotes average superimposed on individual trials (black). (right, top) Individual trials raster plot of spiking with 1 s constant laser stimulation of TRN terminals in striatum. Blue window represents stimulation period. (right, bottom) Perievent time histograms of spiking (PETH) with 1 s constant laser stimulation of TRN terminals in striatum.

To further test the functional connectivity of this projection from TRN to striatal PV interneurons, whole-cell voltage clamp recordings from PV interneurons following TRN terminal stimulation were conducted (Figure 3g). Brief paired blue laser stimulation of TRN ChR2-positive terminals in the striatum over PV interneurons, in the presence of AMPAR and NMDAR antagonists, exhibited fast latency, monosynaptic IPSCs in 53.8% (7/13) of the cells recorded (Figure 3h–j). These large, reliable, and paired-pulse depressing IPSCs were blocked by the GABA_A_R antagonist picrotoxin (Figure 3h). Connectivity was not determined by location as PV interneurons in close proximity to other connected PV interneurons did not show connectivity in the same brain slice. Considering potential subtypes of PV interneurons in striatum(Garas et al., 2016), we compared resting membrane properties including capacitance, membrane potential, tau and holding current between TRN connected and non-connected PV interneurons, and no significant differences were observed (Figure 3—figure supplement 1a–d). In order to test the selectivity of this input, neighboring PV-negative cells (putative SPNs) were also recorded, yet none of these neurons exhibited any functional connectivity (0/8 cells) (Figure 3i). In current clamp, one second constant blue laser stimulation of TRN axon terminals was sufficient to suppress current injection-evoked spiking in PV interneurons (Figure 3k). These results are consistent with the viral tracing data and suggest a selective, functional TRN inhibitory input to striatal PV interneurons.

To determine if the TRN is sufficient to inhibit PV interneurons and affect basal ganglia outputs *in vivo*, optogenetic stimulation of TRN terminals in the striatum was paired with extracellular neuronal recordings using a multi-electrode array (Figure 3l, see Figure 3—figure supplement 2 for array placement) (Howard et al., 2017; Jin et al., 2014). Of the over two hundred putative SPNs recorded, around 40% show a firing rate change during light stimulation (83/205). Noticeably, half of the responsive SPN population (44/83) exhibited delayed inhibition during the one second constant laser stimulation (Figure 3m,o) while others (39/83) exhibited slow latency excitation (Figure 3n,o). While the firing modulation observed in SPNs represents the net network effects of TRN stimulation on basal ganglia output, potential disinhibition of SPNs via TRN inhibition of striatal PV interneurons may contribute to some of these effects. Indeed, four putative striatal PV interneurons (fast-spiking interneurons) were identified in our recordings and all of them show inhibited firing activity during TRN terminal stimulation (Figure 3—figure supplement 3). Overall, TRN is capable of functionally regulating striatal PV interneurons and affecting basal ganglia output *in vivo*.

### GPe functional targeting of striatal ChAT and PV interneurons

While previous studies focused on the excitatory inputs to striatum (Ding et al., 2010; Lapper and Bolam, 1992), inhibition also plays an important role in controlling striatal activity, especially through interneurons (Koós and Tepper, 1999; Silberberg and Bolam, 2015; Tepper et al., 2010). In addition to the novel inhibitory input to striatum from TRN, we explored another major inhibitory projection to the striatum from the GPe (Bevan et al., 1998; Gittis et al., 2014; Mallet et al., 2012; Saunders et al., 2016). Rabies tracing revealed that all basal ganglia nuclei similarly innervate both ChAT and PV interneurons, and of these basal ganglia inputs, GPe was the most predominant (two-way ANOVA, *F*_(6, 63)_=27.27, p<0.0001, Figure 4—figure supplement 1a). Previous studies have suggested that GPe primarily targets PV interneurons and SPNs (Bevan et al., 1998; Gittis et al., 2014; Mallet et al., 2012; Saunders et al., 2016) along with evidence of an anatomical connection to ChAT interneurons (Guo et al., 2015; Mallet et al., 2012). However, our rabies tracing results found that similar proportions of GPe neurons project to both striatal ChAT and PV interneurons (Figure 4—figure supplement 1a). To begin to characterize this GPe projection to ChAT and PV interneurons, rabies-labeled GPe brain sections were immunohistochemically stained for different cell markers (Hernández et al., 2015; Mallet et al., 2016), and few rabies-eGFP GPe neurons were colocalized with either PV immmunostaining (ChAT-projecting: 3.5%, PV-projecting: 2.8%; Figure 4—figure supplement 1b–e) or ChAT immmunostaining (ChAT-projecting: 1.6%, PV-projecting: 1.3%; Figure 4—figure supplement 1c,d). Consistent with previous studies, transcription factors NPAS1 and FoxP2, which are enriched in pallidostriatal projection neurons (Glajch et al., 2016; Hernández et al., 2015), colocalized with a large population of retrogradely labeled eGFP-positive rabies neurons. Rabies eGFP expression for striatal ChAT or PV-projecting GPe neurons colocalized similarly with all immunohistochemical markers tested (Figure 4—figure supplement 1c). These data suggest that ChAT and PV interneurons receive comparable numbers of input neurons from potentially overlapping populations of GPe cell types.

**Figure 4.**
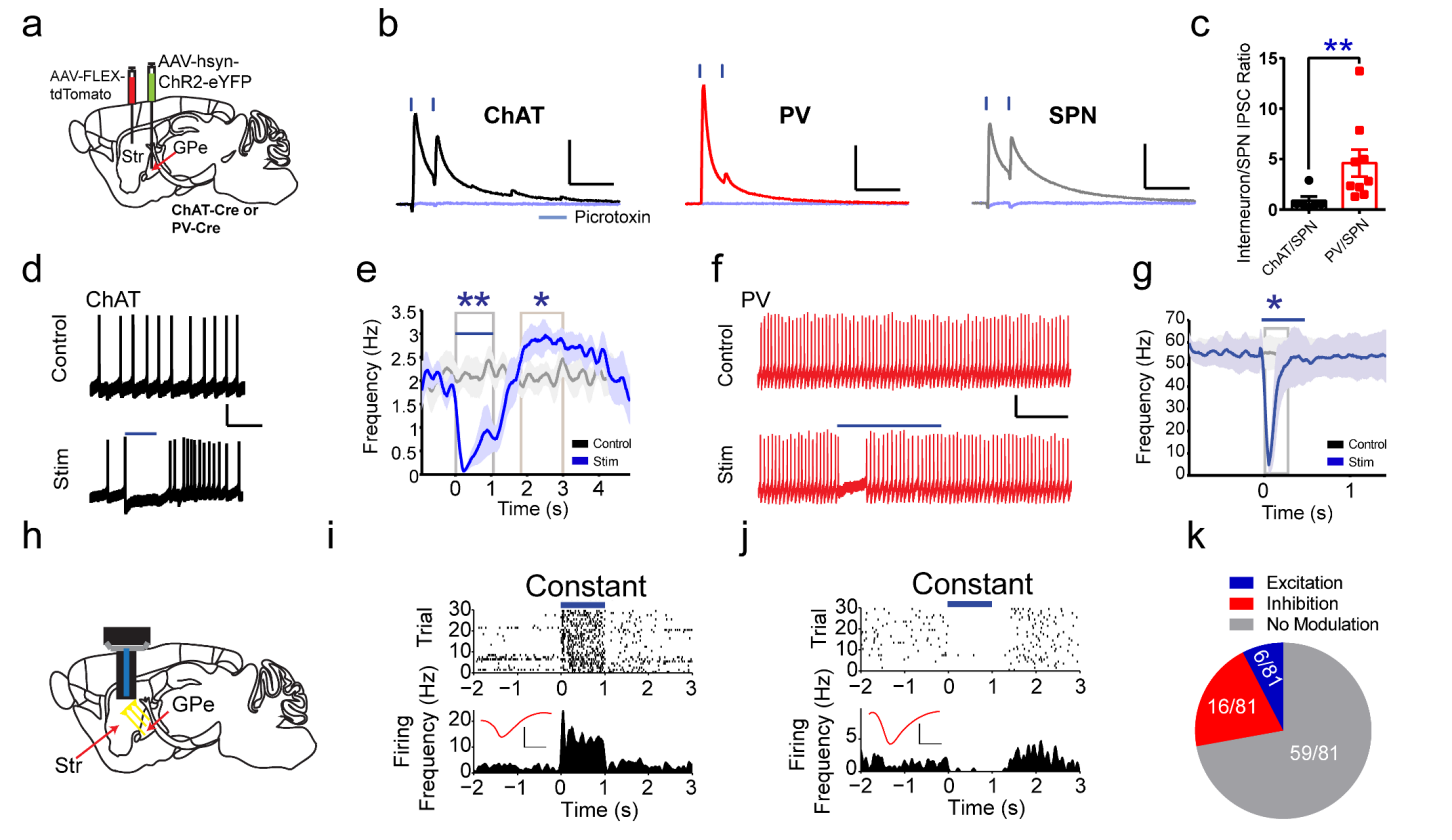
Functional projections of GPe to striatal ChAT and PV interneurons. (**a**) Experimental approach to label GPe inputs with ChR2 and express tdTomato in ChAT or PV interneurons. (**b**) Averaged paired-pulse (50 ms ISI) light-evoked IPSCs in striatal ChAT interneuron (black), PV interneuron (red) and SPN (grey) following GPe terminal stimulation in striatum. Cells are held at −10 mV in the presence of AMPAR (10 µM CNQX) and NMDAR (50 µM DL-APV) antagonists. IPSCs are blocked by the GABA_A_R antagonist picrotoxin (100 µM) (light blue trace). Scale bars, 100 ms, 200 pA. See Figure 4—figure supplement 2 for detailed quantification. (**c**) The ChAT/SPN paired IPSC ratio is lesser than the PV/SPN paired IPSC ratio (Mann Whitney Test, p=0.0076). (**d–e**) ChAT interneuron tonic firing is paused by light-evoked stimulation of striatal GPe terminals, evident from single trace (**d**) and the firing rate PETH (**e**). Scale bars, 1 s, 20 mV. Dark line represents mean and shading denotes SEM in PETH. GPe terminal stimulation promotes ChAT tonic firing activity pause (two-tailed t-test, p=0.0014) and subsequent rebound (two-tailed t-test, p=0.0469). See Figure 4—figure supplement 2h–j for multiple trial raster and quantification. (**f–g**) Suppression of spiking activity in striatal PV interneurons following GPe terminal stimulation for single trace (**f**), and the firing rate PETH (**g**). Scale bars, 250 ms, 25 pA. Quantification of suppression of PV interneuron spiking activity by GPe terminal stimulation (two-tailed t-test, p=0.0401). See Figure 4—figure supplement 2k,l for multiple trial raster and quantification. (**h**) *In vivo* recording of striatal SPNs during optogenetic stimulation of GPe terminals in striatum. (**i–j**) Representative SPNs showing excitation (**i**) or inhibition (**j**) during optogenetic GPe terminal stimulation. Inset (red): Average single unit waveforms of a putative SPN. Scale bars 0.2 ms, 50µV. (**k**) Pie chart of light modulated SPNs firing activity following GPe laser stimulation. The following figure supplements are available for Figure 4: Figure 4—figure supplements 1 and 2.

To functionally confirm the GPe to striatal connection, pairs of ChAT interneurons and neighboring SPNs were recorded in voltage clamp with brief laser stimulation of ChR2-expressing GPe terminals in striatum. Fast latency, monosynaptic IPSCs were observed in both ChAT-positive (11/11) and ChAT-negative neurons (putative SPNs, 7/7) (Figure 4a,b). These IPSCs were confirmed by blockade with the GABA_A_R antagonist picrotoxin (Figure 4b). IPSC amplitudes and probability of release from neighboring ChAT and SPNs were similar (Figure 4—figure supplement 2a–c), but SPNs had increased inverse of the coefficient of variation squared (1/(CV)^^2^) (Figure 4—figure supplement 2d, Mann Whitney, p=0.0114), a presynaptic measure suggesting increased number or function of inhibitory synapses on SPNs. Recordings from pairs of PV interneurons and neighboring SPNs revealed that PV interneurons exhibited larger IPSCs (two-tailed t-test, p<0.0001), higher release probability (two-tailed t-test, p=0.0114), and greater 1/(CV)^^2^ (two-tailed t-test, p=0.0277) (Figure 4b, Figure 4—figure supplement 2e–g), suggesting GPe inhibitory presynaptic release and/or number of synapses is greater on PV interneurons than on SPNs (Bevan et al., 1998; Gittis et al., 2014; Glajch et al., 2016; Mallet et al., 2012; Saunders et al., 2016). Overall, the ChAT to SPN IPSC ratio was lower than the PV to SPN ratio, suggesting increased functional connectivity from GPe to PV compared to ChAT interneurons, despite similar numbers of GPe neurons projecting to both (Figure 4c, Mann Whitney test, p=0.0076).

**Figure 4—figure supplement 1.**
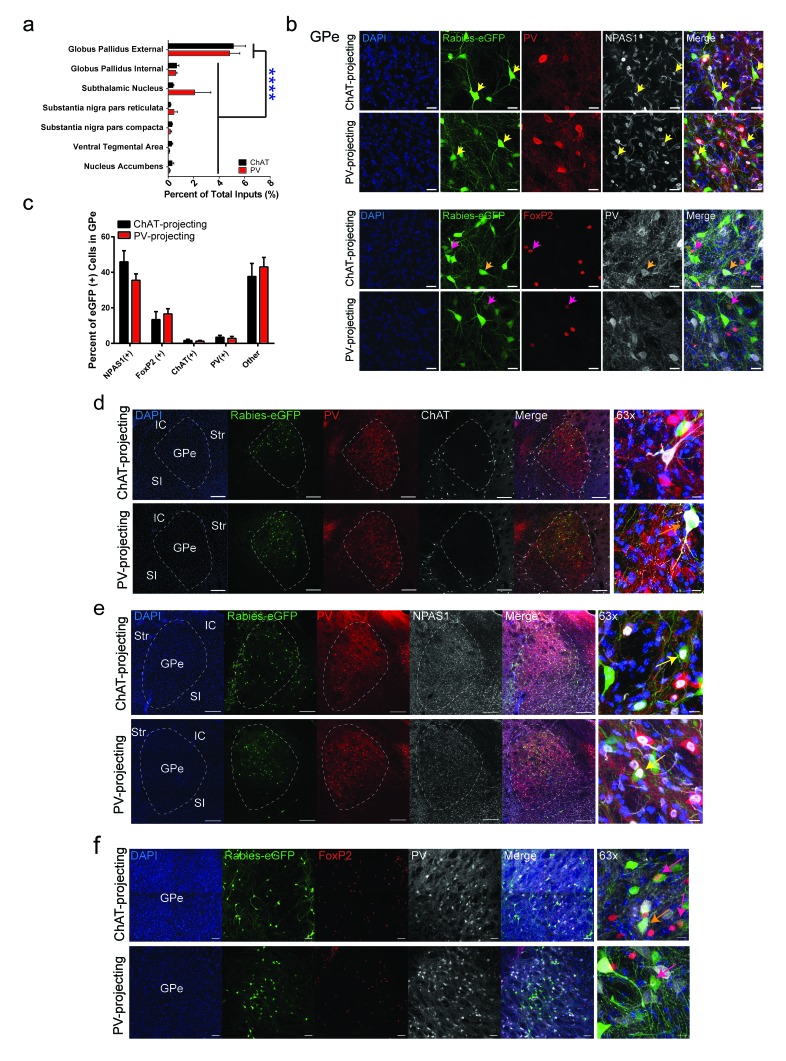
Cell-type identity of GPe projections to striatal ChAT and PV interneurons. (**a**) GPe is the predominant basal ganglia input to ChAT and PV interneurons (two-way ANOVA, F _(6,63)_=27.27, p<0.0001). (**b–c**) (top) Few GPe rabies-eGFP neurons colocalize with immunostaining for PV, while a population of GPe rabies-eGFP neurons colocalized with the transcription factor, (top) NPAS1 or (bottom) FoxP2. Scale bars, 20 µm. Yellow arrowheads denote rabies-eGFP and NPAS1 colocalized neurons. Pink arrowheads denote rabies-eGFP and FoxP2 colocalized neurons. Orange arrowheads denote rabies-eGFP and PV colocalized neurons. (**d**) Few GPe rabies-eGFP neurons colocalize with immunostaining for PV and ChAT. Orange arrows denote colocalized rabies-eGFP expression with ChAT. IC, internal capsule; Str, striatum; GPe, globus pallidus external segment; SI, substantia innominata. Scale bars, 200 µm.; 63x scale, 20 µm. (**e**) A population of GPe rabies-eGFP neurons colocalize with the transcription factor NPAS1. Yellow arrows denote colocalized rabies-eGFP expression with NPAS1 immunostaining. Scale bars, 200 µm; 63x scale, 20 µm. (**f**) A population of rabies-eGFP GPe neurons colocalize with FoxP2 immunostaining. Pink arrows denote colocalized rabies-eGFP expression with FoxP2 immunostaining, while orange arrows denote colocalized rabies-eGFP and PV immunostaining. Scale bar, 50 µm; 63x scale, 10 µm.

**Figure 4—figure supplement 2.**
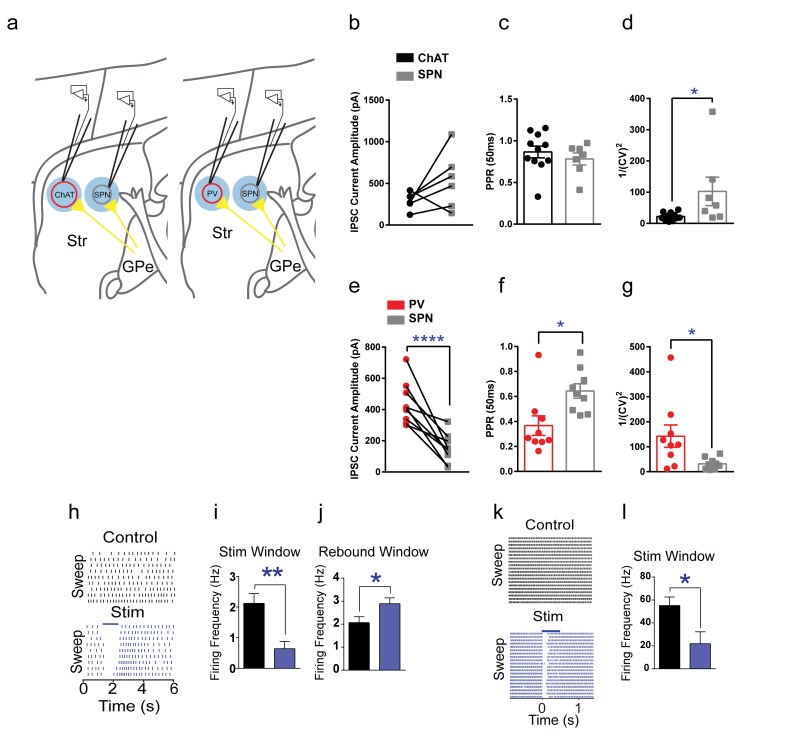
GPe functional inhibitory connectivity to ChAT interneurons, PV interneurons and SPNs. (**a**) Cartoon schematic showing whole-cell recordings from pairs of interneurons and neighboring SPNs following light-evoked GPe terminal stimulation. (**b**) IPSCs from pairs of tdTomato (+) ChAT interneurons and neighboring tdTomato (-) SPNs. (**c**) Paired-pulse ratio (ISI, 50 ms) of ChAT and SPNs IPSCs. (**d**) 1/(CV)^2^ measure of trial-to-trial IPSC amplitude variance is larger in SPNs compared to ChAT (Mann Whitney test, p=0.0114). (**e**) PV IPSC amplitude is significantly larger than paired neighboring SPNs (two-tailed t-test, p<0.0001). (**f**) Significantly reduced paired-pulse ratio PV IPSCs (50 ms) versus paired neighboring SPNs suggests an increased probability of release (two-tailed t-test, p=0.0114). (**g**) 1/(CV)^2^ measure of trial-to-trial IPSC amplitude variance is larger in PV versus SPNs (two-tailed t-test, p=0.014). (**h**) ChAT interneuron tonic firing is paused by light-evoked stimulation of striatal GPe terminals, evident over multiple trials. (**i–j**) Statistic results of GPe terminal stimulation on ChAT firing activity pause (i, two-tailed t-test, p=0.0014) and rebound (j, two-tailed t-test, p=0.0469). (**k**) Suppression of spiking activity in striatal PV interneurons following GPe terminal stimulation over multiple trials. (**l**) Quantification of suppression of PV interneuron spiking activity by GPe terminal stimulation (two-tailed t-test, p=0.0401).

Pauses in ChAT interneuron firing are associated with cues that predict reward (Kimura et al., 1984), so we next tested if these inhibitory GPe inputs to striatum are capable of suppressing tonic ChAT firing. In whole-cell current clamp recordings, ChAT interneurons were tonically active and rested at modestly depolarized potentials (~ −60 mV, Figure 4d). Interleaved blue laser light stimulation of GPe inhibitory terminals on ChAT interneurons was sufficient to significantly reduce tonic firing (Figure 4d, Figure 4—figure supplement 2h–j, two-tailed t-test, p=0.0043). After cessation of laser stimulation, ChAT neurons showed significant rebound activity spiking at rates above baseline (Figure 4e, Figure 4—figure supplement 2h–j, two-tailed t-test, p=0.0469). Furthermore, inhibitory inputs from GPe were sufficient to inhibit current injection-induced spiking in PV interneurons for a few hundred milliseconds following blue laser stimulation (Figure 4f,g; Figure 4—figure supplement 2k,l; two-tailed t-test, p=0.0401). These data suggest that SPNs, striatal ChAT and PV interneurons all receive inhibitory inputs from GPe, which is sufficient to generate pause-burst activity in ChAT interneurons and suppression of spiking in PV interneurons. To further explore the net functional effects of GPe to striatum projections *in vivo*, extracellular recordings paired with optogenetic stimulation of ChR2-expressing GPe terminals in striatum were performed (Figure 4h). It revealed that of all the SPNs responsive to GPe terminal stimulation (22/81), the majority showed inhibited firing activity (16/22) while a small proportion was excited (6/22) (Figure 4i–k). These results suggest that GPe sends a prominent inhibitory input to both striatal ChAT and PV interneurons and exert powerful feedback control on striatal activity.

### Excitatory pedunculopontine nucleus projections to striatal ChAT interneurons

**Figure 5.**
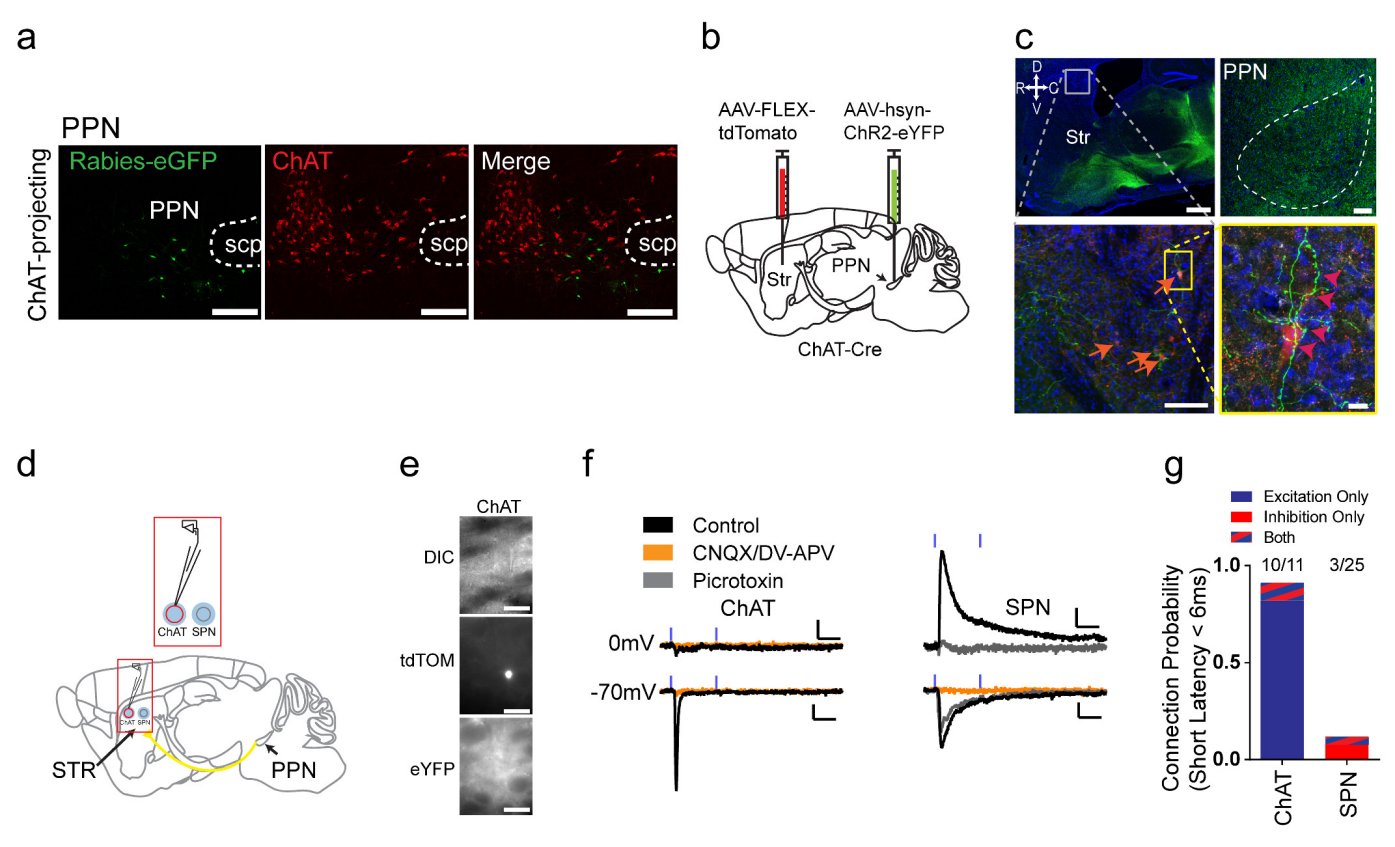
Excitatory projections from pedunculopontine nucleus to striatal ChAT interneurons. (**a**) Rabies-labeled projections to striatal ChAT interneurons from the PPN are not cholinergic. Note no colocalized eGFP (green) and ChAT immunostaining (red). scp, superior cerebellar peduncle. Scale bars, 200 µm. (**b**) Viral injection scheme to anterogradely verify the PPN projections to striatum. (**c**) (left) Sagittal image of eYFP terminal expression following injection of AAV-ChR2-eYFP in PPN. Scale bar, 1 mm (top). (right) Sagittal image of PPN injection site with AAV-ChR2-eYFP expression. Scale bar, 100 µm. Note many eYFP fibers from PPN target on tdTomato expressing ChAT interneurons (orange and pink arrowheads). Scale bar, 200 µm (left), 20 µm (right). (**d**) Diagram for functional validation of PPN projections to striatum in brain slices. (**e**) DIC and epifluorescent images of patched dorsal striatal ChAT and neighboring SPN neurons. Scale bar, 200 µm (DIC), 50 µm (epifluorescent). (**f**) Example average current trace at −70 mV or 0 mV under control (black), after blocking AMPAR and NMDAR currents (yellow) or blocking GABA_A_R currents (grey). Scale bar, 25 ms, 25 pA. (**g**) Percentage of ChAT and SPN neurons exhibiting fast latency (<6 ms) excitatory (blue), inhibitory (red) or both (blue/red stripes) currents following PPN terminal stimulation.

The pedunculopontine nucleus (PPN), a part of the ascending reticular activating system, has been observed to project directly to the striatum (Dautan et al., 2014); yet, the cell type and physiology of this pathway has not been fully elucidated. Notably, in the rabies tracing data, PPN had a noticeable bias to ChAT interneurons, compared to other subcortical projections (two-way ANOVA, *F*_(1, 72)_=6.034, p=0.0008). PPN projections to striatum were previously thought to be cholinergic (Dautan et al., 2014). However, no overlap in immunostaining for ChAT and eGFP rabies-labeled neurons in the PPN was observed (Figure 5a). To begin to characterize this long-range connection, non-Cre dependent AAV-ChR2 was injected into PPN and terminals were observed innervating all basal ganglia nuclei, including striatum (Figure 5b). Interestingly, while the eGFP axon terminal expression in the striatum was sparse, it was directed, wrapping the cell somas and proximal dendrites of ChAT interneurons (Figure 5c). Overall these data suggest that PPN sends a non-cholinergic projection to striatum that preferentially targets ChAT interneurons.

To functionally explore this input and confirm the anatomical findings, whole-cell voltage clamp recordings of ChAT interneurons combined with optogenetic stimulation of PPN terminals was used to determine connectivity and input type (Figure 5d–f). PPN is a heterogeneous hindbrain nucleus composed of cholinergic, glutamatergic and GABAergic neurons (Martinez-Gonzalez et al., 2011; Mena-Segovia et al., 2009). Recordings at the reversal potential for excitatory and inhibitory transmission before and after the addition of AMPAR/NMDAR antagonists or GABA_A_R antagonists was used to determine if PPN axon terminals are excitatory or inhibitory, respectively (Figure 5f). ChAT interneurons exhibited fast latency, monosynaptic (<6 ms), excitatory currents in a vast majority of neurons sampled (10/11), while only a few neighboring SPNs (3/25) exhibited fast latency (<6 ms), excitatory or inhibitory connections (Figure 5g). Notably, the current experiments do not allow us to disambiguate neurotransmitter co-release from a single terminal or differing classes of PPN inputs on a single striatal interneuron (Wang and Morales, 2009). Overall, these results suggest that different from SPNs, ChAT interneurons receive strong functional excitatory inputs on the soma and proximal dendrites from PPN.

## Discussion

### Differential excitatory inputs to ChAT and PV striatal interneurons

While some hints at specifying afferent inputs to ChAT and PV interneurons have been observed with anterograde tracers or ultrastructural studies (Chang and Kita, 1992; Gonzales et al., 2013; Mallet et al., 2005; Mallet et al., 2012; Ramanathan et al., 2002), more comprehensive information on the brain-wide inputs are needed for quantitative comparisons and providing further insights into their functional roles in behavior. Our tracing results suggest that the majority of excitatory inputs to both ChAT and PV interneurons come from the cerebral cortex. Our data confirm previous reports that striatal PV interneurons receive substantial inputs from M1 and S1 (Parthasarathy and Graybiel, 1997; Ramanathan et al., 2002), suggesting a role for PV interneurons in sensorimotor integration. In contrast, we found that projections from associative regions of cortex, including the orbital and cingulate cortex, and the associative regions of the thalamus, including intralaminar nuclei and mediodorsal nucleus, preferentially targeted ChAT interneurons. The orbital cortex has been shown to be involved in decision making, reversal learning and shifting from goal-directed to habit learning (Gourley et al., 2013; Gremel and Costa, 2013; McAlonan and Brown, 2003). The cingulate cortex has been described as an interface between motivation, cognition and action with roles associated with effort, action value, reward expectancy, and behavioral flexibility (Cowen et al., 2012; Hadland et al., 2003; Hayden and Platt, 2010; Johnston et al., 2007; Paus, 2001; Shidara and Richmond, 2002; Walton et al., 2004). The thalamic intralaminar nuclei and mediodorsal nucleus are involved in attention and cognitive processing of information (Kimura et al., 2004; Minamimoto and Kimura, 2002; Parnaudeau et al., 2013). This preferential connectivity suggests a specific role of ChAT interneurons in context-dependent modulation of action and behavioral flexibility (Matamales et al., 2016). Indeed, recent work suggests cholinergic interneurons use orbitofrontal inputs to track current environmental state during behavior (Stalnaker et al., 2016). Furthermore, we find a novel, functional excitatory pathway from the PPN to striatal ChAT interneurons. This pathway, part of the ascending reticular activating system, may coordinate with the basal forebrain to regulate levels of arousal in cortex and striatum concomitantly, representing a mechanism of context-dependent gain control of behavior (see Figure 2—figure supplement 2 for visual anatomical summary of ChAT and PV inputs).

When our current data are considered collectively with previous studies using monosynaptic rabies tracing from other striatal cell types (Smith et al., 2016; Wall et al., 2013), a general theme appears. Each cell type in the striatum appears to have access to the same inputs within a topographic domain (Hintiryan et al., 2016), but significant preferences do exist for certain cell types, which imbues functional distinctions. In the case of direct and indirect pathway SPNs, it was found that the amygdala almost exclusively targeted direct pathway, but not indirect pathway SPNs (Wall et al., 2013). For the patch/matrix SPNs, the most prominent distinction was limbic subcortical inputs, primarily from the bed nucleus of the stria terminalis preferentially innervating the patch compartment (Smith et al., 2016). For ChAT and PV interneurons, our rabies tracing indicates a number of differences that lend insight into the differential role these interneuron subtypes in regulating striatal output to control action selection and learning. Interestingly, when analyzing the cortical laminar breakdown in associative versus sensorimotor cortices we observed more superficial layer projections from associative cortex versus more deep layer projections from sensorimotor cortex in both the ChAT and PV rabies tracing dataset, which was also observed in the patch/matrix rabies tracing data set (Smith et al., 2016). This laminar organization may be a general organizing principle of the cortical inputs to the dorsal striatum.

### A TRN to PV interneuron inhibitory circuit

Most previous studies have focused on the excitatory inputs to striatum (Ding et al., 2008; Ding et al., 2010). Here we report a novel, GABAergic input from the TRN that projects to striatal PV interneurons. This reliable, short latency TRN-PV pathway was sufficient to inhibit PV interneuron spiking and alter SPN activity *in vivo*. However, additional interneuron subtypes will need to be tested to determine the precise mechanism of TRN-mediated SPN modulation. Traditionally, TRN is thought to send projections only within thalamus regulating thalamocortical communications and acting as an attentional spotlight or filter (Pinault, 2004). In these models, when one sensory modality is active during attention, a winner-take-all network between thalamus-TRN reciprocal connections would inhibit the other modality and facilitate information processing in the attended domain.

The projection from TRN to striatal PV interneurons found in our current study was first identified through rabies tracing experiments. This projection represents a substantial and selective inhibitory input to striatal PV interneurons as this pathway has not been observed in previous rabies tracing datasets on other striatal cell types (Guo et al., 2015; Smith et al., 2016; Wall et al., 2013). This pathway was further verified independently by anterograde and intersectional tracing experiments in two separate mouse lines along with slice electrophysiology combined with optogenetics.

The function of this TRN-striatal PV interneuron pathway could be particularly significant. Activation of TRN inputs would inhibit PV interneuron activity and potentially excite SPNs via disinhibition. Our electrophysiological recordings from brain slices detected large IPSCs in more than half of striatal PV interneurons sampled. Using *in vivo* striatal recordings with optogenetic stimulation of TRN terminals in striatum, we observed that putative striatal fast spiking interneurons (FSIs) were inhibited during optical stimulation, while a subpopulation of SPNs showed firing rate modulation either directly via PV interneurons or indirectly through a network effect. This subcortical pathway is well positioned to relay attention-related information directly to the striatum. By targeting striatal PV interneurons, we speculate that it might be possible to effectively bias action selection and coordinate an animal’s ongoing behavior with corresponding attentional state (Berke, 2011; Gage et al., 2010; Jin et al., 2014). Potentially, this pathway could serve as a subcortical bridge for promptly coordinating sensory attention and actions.

### A GPe to ChAT interneuron inhibitory circuit

Previous data suggested that GPe projected to striatal PV interneurons and SPNs (Bevan et al., 1998; Mallet et al., 2012) with some anatomical evidence of a projection to ChAT interneurons (Guo et al., 2015; Mallet et al., 2012). Our rabies tracing data confirmed these connections as well as demonstrated for the first time a functional connection from GPe to striatal ChAT interneurons. The GPe projections to striatal ChAT and PV interneurons draw from similar proportions of NPAS1, FoxP2 and a small minority of PV and ChAT neurons. However, we did observe a population of rabies-eGFP neurons that did not colocalize with any of the immunohistochemical markers, potentially suggesting another subtype such as Lhx6-expressing GPe neurons (Mastro et al., 2014). Utilizing whole-cell recordings, we confirmed that the GPe has a high degree of connectivity to both striatal ChAT and PV interneurons, as well as SPNs. In classical Pavlovian conditioning or stimulus-response learning, ChAT interneurons, which are tonically active, exhibits a pause in firing within a few hundred milliseconds after the presentation of the conditioned stimuli (Aosaki et al., 1994; Kimura et al., 1984; Ravel et al., 1999). This pause response depends on learning and is seen with stimuli predicting both rewarding and aversive outcomes (Ravel et al., 1999). While the precise function of the pause response in ChAT interneurons remains to be elucidated at a behavioral level, it has been suggested to encode the salience of external stimuli and regulate local dopamine release in striatum. The neural mechanism underlying the pause has caused an extensive debate in the field. Several cellular mechanisms involving multiple sources have been proposed, including the contributions from the intrinsic cell membrane properties of ChAT interneurons, dopamine regulation or GABA co-release, and thalamic inputs with the involvement of an unidentified type of striatal GABAergic interneuron (English et al., 2012; Nelson et al., 2014; Schulz and Reynolds, 2013; Straub et al., 2014; Sullivan et al., 2008; Threlfell et al., 2012). The abovementioned mechanisms are not necessarily mutually exclusive, and each might actually contribute to the pause response to a different degree or under various conditions.

In our study, we identified a novel extrastriatal GABAergic input from GPe to ChAT interneurons that is sufficient to suppress tonic firing and generate pause responses similar to those observed in behavior (Aosaki et al., 1994). Considering recent studies implying that striatum-projecting GPe neurons play a crucial role during action cancelling (Mallet et al., 2016), our current results thus further reinforce that the GPe to striatal interneuron projections may be an important part of the feedback circuitry involved in context-dependent action control.

### An excitatory pedunculopontine nucleus input to striatal ChAT interneurons

In this study, we identified a novel, functional excitatory PPN pathway that was selectively enriched in it projections to ChAT interneurons, but not SPNs. Previous literature suggested a significant anatomical cholinergic PPN input to striatum (Dautan et al., 2014). Co-release of glutamate from cholinergic terminals in striatum on ChAT interneurons may be one possible source of this excitatory signal. Yet, previous evidence does not lend strong support for the idea of co-release with a vast majority of cholinergic neurons in PPN lacking expression of vGluT2 mRNA (Wang and Morales, 2009). Alternatively, the lack of rabies labeling of cholinergic cells in PPN may be attributed to a technical limitation with rabies labeling of neuromodulatory synapses. For example, several striatal rabies tracing studies using the same technique do not find significant rabies labeling of dopamine neurons in the substania nigra pars compacta (SNc) (Smith et al., 2016; Wall et al., 2013), despite the dense innervation of dopamine terminals from the SNc in dorsal striatum. Alternatively, ChAT interneurons may not receive direct cholinergic projections from PPN. Additional, studies will be needed to address the role of two sources of acetylcholine from ChAT interneurons or PPN terminals in striatum and determine what, if any, crosstalk occurs on striatal interneurons.

Our anatomical tracing data indicate that ChAT interneurons receive more projections from associative regions of cortex and thalamus (e.g. lateral orbital cortex, retrosplenial cortex, mediodorsal thalamus, etc.). Conversely, PV interneurons receive more inputs from sensorimotor regions, including primary motor and somatosensory cortical areas. These preferential inputs suggest a potential functional distinction for their role in controlling behavior, whereby ChAT interneurons may receive internal cognitive or external environmental information for context-dependent action modulation, while PV interneurons may integrate sensorimotor information for action learning and selection. Together, these data indicate a comprehensive revision of striatal circuitry with distinct, yet complimentary roles for striatal ChAT and PV interneurons in shaping striatal function.

## Materials and methods

**Key resources table:** 

Reagent type (species) or resource	Designation	Source or reference	Identifiers	Additional information
Strain, strain background (*Mus musculus)*	ChAT-IRES-Cre	Jackson Labs	stock #006410; RRID:IMSR_JAX:006410	maintained on a C57BL6/J background
Strain, strain background (*Mus musculus*)	PV-Cre	Jackson Labs	stock #008069; RRID:IMSR_JAX:008069	maintained on a C57BL6/J background
Strain, strain background (*Mus musculus)*	Sst-IRES-Cre	Jackson Labs	stock #028864; RRID:IMSR_JAX:028864	maintained on a C57BL6/J background
Strain, strain background (*Adeno-associated virus)*	AAV5-TVA-mCherry	UNC Viral Vector Core	RRID: SCR_002448	3–4.3 × 10^12^ particles/mL
Strain, strain background (*Adeno-associated virus)*	AAV8-CA-RG	UNC Viral Vector Core	RRID: SCR_002448	1.2–4.3 × 10^12^ particles/mL
Strain, strain background (*Adeno-associated virus)*	(EnvA) SAD-∆G Rabies-eGFP	Salk Vector Core	RRID: SCR_014847	1.6–6.55 × 10^8^ particles/mL
Strain, strain background (*Adeno-associated virus)*	AAV9-Ef1a-DIO-ChR2 (H134R)-eYFP	University of Penn Viral Vector Core	RRID: SCR_015406	two × 10^12^ particles/mL
Strain, strain background (*Adeno-associated virus)*	AAV9-FLEX-tdTomato	University of Penn Viral Vector Core	RRID: SCR_015406	two × 10^12^ particles/mL
Strain, strain background (*Adeno-associated virus)*	AAVretro-EF1a-Flp	UNC Viral Vector Core	RRID: SCR_002448	two × 10^12^ particles/mL
Strain, strain background (*Adeno-associated virus)*	AAV9-hsyn-Con-Fon-hChR2-eYFP	UNC Viral Vector Core	RRID: SCR_002448	two × 10^12^ particles/mL
Strain, strain background (*Adeno-associated virus)*	AAV9-FLEX-eGFP	University of Penn Viral Vector Core	RRID: SCR_015406	two × 10^12^ particles/mL
Strain, strain background (*Adeno-associated virus)*	AAV9-hsyn-ChR2-eYFP	University of Penn Viral Vector Core	RRID: SCR_015406	two × 10^12^ particles/mL
Antibody	anti-PV (mouse,monoclonal)	MilliporeSigma	P3088; RRID: AB_477329	1/1000
Antibody	anti-PV (rabbit, polyclonal)	Abcam	ab11427; RRID: AB_298032	1/1000
Antibody	anti-ChAT (goat, polyclonal)	MilliporeSigma	AB144P; RRID: AB_2079751	1/100
Antibody	anti-NPAS1 (rabbit, polyclonal)	GeneTex	GTX105876; RRID: AB_424768	1/500
Antibody	anti-mCherry (mouse, monoclonal)	Takara Bio USA, Inc (Clontech Labs)	632543; RRID: AB_2307319	1/250
Antibody	anti-eGFP (rabbit, polyclonal)	Thermo Fisher Scientific	A11122; RRID: AB_1074875	1/400
Antibody	anti-somatostatin (rabbit, polyclonal)	Protos Biotech Corp	NP106SST	1/300
Antibody	anti-FoxP2 (rabbit, polyclonal)	Sigma	HPA000382; RRID: AB_1078908	1/1000
Antibody	donkey anti-mouse 488 (secondary)	Jackson Immunoresearch	715-545-150; RRID: AB_2340846	1/250
Antibody	donkey anti-mouse CY3 (secondary)	Jackson Immunoresearch	715-165-150; RRID: AB_2340813	1/250
Antibody	donkey anti-mouse CY5 (secondary)	Jackson Immunoresearch	715-175-150; RRID: AB_2340819	1/250
Antibody	donkey anti-rabbit 488 (secondary)	Jackson Immunoresearch	711-545-152; RRID: AB_2313584	1/250
Antibody	donkey anti-rabbit CY3 (secondary)	Jackson Immunoresearch	711-165-152; RRID: AB_2307443	1/250
Antibody	donkey anti-rabbit CY5 (secondary)	Jackson Immunoresearch	711-175-152; RRID: AB_2340607	1/250
Antibody	donkey anti-goat 488 (secondary)	Jackson Immunoresearch	705-545-147; RRID: AB_2336933	1/250
Antibody	donkey anti-goat CY3 (secondary)	Jackson Immunoresearch	705-165-147; RRID: AB_2307351	1/250
Antibody	donkey anti-goat CY5 (secondary)	Jackson Immunoresearch	705-175-147; RRID: AB_2340415	1/250
Chemical compound, drug	NBQX disodium salt hydrate	MilliporeSigma	N183	10 uM (final)
Chemical compound, drug	DL-APV	MilliporeSigma	A5282	50 uM (final)
Chemical compound, drug	Picrotoxin	MilliporeSigma	P1675	50–100 uM (final)
Chemical compound, drug	NMDG	MilliporeSigma	M2004	
Chemical compound, drug	HCl	MilliporeSigma	H1758	
Chemical compound, drug	KCl	MilliporeSigma	P9541	
Chemical compound, drug	NaH2PO4	MilliporeSigma	S3139	
Chemical compound, drug	NaHCO3	MilliporeSigma	S6014	
Chemical compound, drug	Glucose	MilliporeSigma	G5767	
Chemical compound, drug	Sodium L-Ascorbate	MilliporeSigma	A4034	
Chemical compound, drug	Sodium Pyruvate	MilliporeSigma	P2256	
Chemical compound, drug	Thiourea	MilliporeSigma	T8656	
Chemical compound, drug	MgSO4	MilliporeSigma	M2643	
Chemical compound, drug	CaCl2	MilliporeSigma	223506	
Chemical compound, drug	MgCl2	MilliporeSigma	M9272	
Chemical compound, drug	KMeSO4	MilliporeSigma	83000	
Chemical compound, drug	HEPES	MilliporeSigma	H4034	
Chemical compound, drug	EGTA	MilliporeSigma	3777	
Chemical compound, drug	Mg-ATP	MilliporeSigma	A9187	
Chemical compound, drug	Na-GTP	MilliporeSigma	G8877	
Chemical compound, drug	CsCl	MilliporeSigma	C4036	
Chemical compound, drug	CsMeSO3	MilliporeSigma	C1426	
Chemical compound, drug	QX-314	MilliporeSigma	L5783	
Chemical compound, drug	TEA-Cl	MilliporeSigma	T2265	
Software, algorithm	MATLAB		RRID: SCR_001622	
Software, algorithm	GraphPad Prism 6		RRID: SCR_002798	
Software, algorithm	Adobe Illustrator CS6		RRID: SCR_010279	
Software, algorithm	pClamp9		RRID: SCR_011323	
Software, algorithm	Fiji/Imagej		RRID: SCR_002285	
Other	Allen Reference Atlas		RRID: SCR_013286	

### Delta G-Rabies tracing viral injections

All procedures were approved by the Salk Institute Institutional Animal Care and Use Committee. Group housed male and female adult mice (8–12 weeks) were used in the study. Heterozygous ChAT-IRES-Cre (*Chat)* (Jackson Labs, stock # 006410, RRID:IMSR_JAX:006410) and PV-Cre (*Pvalb*) (Jackson Labs, stock # 008069, RRID:IMSR_JAX:008069) mice were backcrossed to C57Bl6/J (>9 generations). For G-deleted rabies mediated cell tracing, animals were anesthetized with ketamine/xylazine (100 mg/kg/10 mg/kg) and mounted on a stereotaxic device (Kopf Instruments; Tujunga, CA). The skull was leveled at bregma and lambda and a small hole was drilled at the dorsal central striatal coordinate of AP +0.5, ML −1.8. Only the right hemisphere was used for this study. A Hamilton syringe (33 gauge needle) containing freshly mixed AAV5-TVA-mCherry (3–4.3 × 10^12^ particles/mL; UNC Vector Core; Chapel Hill, NC, RRID: SCR_002448) and AAV8-CA-RG (1.2–4.3 × 10^12^ particles/mL; UNC Vector Core; Chapel Hill, NC, RRID: SCR_002448) (total 1 µl) was slowly lowered to a depth of DV −2.25 from the dura. The virus cocktail was injected slowly over 10mins. The needle was left in place for 5 min additional minutes and then the needle was slowly retracted over 5 min to reduce virus moving into the tract. Mice were sutured and returned to their home cage with ibuprofen (50 mg/kg/day) in their drinking water for four days. After three weeks to allow for maximal expression of helper viruses animals are injected with 1.5 µl of (EnvA) SAD-∆G Rabies-eGFP (1.6–6.55 × 10^8^ particles/mL, Salk Vector Core, La Jolla, CA, RRID: SCR_014847) on an angle (18°) to avoid labeling any neurons in the initial injection tract in the same target region. Injection locations were identical in ChAT-Cre and PV-Cre animals suggesting that connectivity differences observed are attributable to interneuron target and not topography. A total of (6) ChAT-Cre and (5) PV-Cre mice (high input expression) were used in tracing experiments and included in analysis following a power analysis. These experiments were performed once and all brains (6 ChAT, 5 PV) were included in the analysis.

### Histology and image analysis

Ten days after rabies injection mice were anesthetized with an overdose of ketamine/xylazine and transcardially perfused with 0.01M PBS (30–40 mL) followed by 4% paraformaldehyde (PFA)/0.1M PB pH 7.4 (30–40 mL) with a peristaltic perfusion pump (Cole Parmer; Vernon Hills, IL). The brain was carefully extracted and post-fixed in 4% PFA overnight (24 hr). The brain was then transferred to 30% sucrose/0.1M PB for 1–2 days until the brain equilibrated and sunk. The brain was then blocked with a brain matrix (Zivic Instruments; Pittsburg, PA) to obtain a true coronal plane and mounted on a freezing microtome. Additional ChAT-Cre and PV-Cre animals were cut in the sagittal plane to avoid missing any targets in rostral or caudal sites. Only neurons ipsilateral to the injection site were quantified. Coronal slices were collected at 50 µm resolution in 96 well plates containing cyroprotectant (0.1M phosphate buffer, ethylene glycol, glycerol) to maintain AP position. Every other brain slice was plated on super frost plus slides (Thermo Fisher Scientific, Waltham, MA) for a whole brain reconstruction at 100 µm resolution. Slides were counterstained with DAPI and cover slipped with aqua-poly mount mounting media (Polysciences, Inc; Warrington, PA). Slides were scanned on an automated slide scanner (Olympus VS120) at 10x in the blue and green channels. Images were batch converted to composite TIFFs and saved for image analysis. Individual channels were thresholded when necessary to better distinguish cells in densely expressing regions. Coronal mouse brain reference atlas images overlays were used from the Allen Mouse Brain Atlas (RRID: SCR_013286) (Website: © 2015 Allen Institute for Brain Science. Allen Mouse Brain Atlas [Internet]. Available from: http://mouse.brain-map.org) and made into to transparent overlays in Adobe Illustrator CS6 (San Jose, CA)(RRID: SCR_010279). De-identified images were placed in Adobe Illustrator CS6 and the proper AP coronal reference image was overlaid on the scanned brain slice. The reference image was warped to match slice boundaries and white matter anatomical landmarks at multiple points. Each cell soma in each brain region and cortical layer was counted and AP plane was recorded. Counters were blind to condition and were vetted with an inter-rater reliability of >95% when counting the same brain before analysis. To check the most rostral and caudal regions of the brain for expression additional mouse brains in the sagittal plane were analyzed. Total number of neurons minus striatal expression was used to determine the total number of input neurons to either ChAT or PV interneurons. All data is presented as the percent of total number of input neurons to normalize for difference in the number of neurons labeled from mouse to mouse.

Higher order association cortex grouping included orbital, prelimbic, insular, cingulate, secondary motor cortex, secondary somatosensory cortex, retrosplenial and posterior parietal cortex. Sensorimotor cortex grouping included primary motor cortex, primary somatosensory cortex as well as sensory regions like auditory cortex and visual cortex. Cortical layer distribution was identified by overlaid brain atlas template aligned to corpus callosum and edge of cortex along with DAPI counter staining. Distinctive layer 4 DAPI staining was also used as a landmark to divide superficial or deep layer expression in certain cortical regions.

For starter cell quantification six slices containing the rostral to caudal striatum and surrounding the injections site were immunostained with anti-mCherry (TVA) and anti-eGFP antibodies. Each image was bisected into dorsal medial and dorsal lateral striatum subdivisions. Total number of TVA-mCherry and rabies-eGFP colocalized starter cells were counted in image j. Data is presented as percent of total starter cells.

### Immunohistochemistry

Rabies-eGFP brain slices were washed 3x in 1X TBS (15 min/each) in net wells on an orbital shaker. Slices were blocked and permeabilized in TBS++ (3% normal horse serum (Jackson ImmunoResearch, West Grove, PA), 0.25% TritonX-100 in 1X TBS) for 1 hr at room temperature on a shaker. Primary antibodies (anti-PV mouse, 1:1000, P3088 (MilliporeSigma, St. Louis, MO; RRID: AB_477329); anti-PV rabbit, 1:1000, ab11427 (Abcam, Eugene, OR; RRID: AB_298032); anti-ChAT goat, 1:100, AB144P (MilliporeSigma, St. Louis, MO; RRID: AB_2079751); anti-NPAS1 rabbit, 1:500, GTX105876 (GeneTex; Irvine, CA; RRID: AB_424768); anti-mCherry mouse 1:100, 632543 (Takara Bio USA, Inc, Mountain View, CA; RRID: AB_2307319); anti-eGFP rabbit, 1:400, A11122 (Thermo Fisher Scientific, Waltham, MA; RRID: AB_1074875); anti-Somatostatin rabbit 1:300, NP106SST, (Protos Biotech Corp, New York, NY); anti-FoxP2 rabbit 1:1000, HPA000382, (Sigma; RRID: AB_1078908)) was diluted in TBS ++and incubated on a shaker at 4°C for 48 hr. Slices are washed 2x in 1X TBS (15 min/each) and 30 min in TBS++. Slices were then incubated in secondary antibody (donkey anti-mouse (RRID: AB_2340846, RRID: AB_2340813, RRID: AB_2340819) or donkey anti-rabbit (RRID: AB_2313584, RRID: AB_2307443, RRID: AB_2340607) or donkey anti-goat (RRID: AB_2336933, RRID: AB_2307351, RRID: AB_2340415) Cy2, Cy3 or Cy5, 1:250, (Jackson ImmunoResearch, West Grove, PA) for 2–3 hr at room temperature on an orbital shaker. Slices were washed three times in 1XTBS (15 min/each), transferred to 0.1M PB, plated on super frost plus slides and cover slipped with aquapoly mount mounting media. Images were collected on a Zeiss 780 confocal microscope with tiling capabilities. Orthogonal projections of the Z-plane of individual channels are presented. Colocalization analysis is performed in Fiji/ImageJ (RRID: SCR_002285). Briefly, individual channels are opened in Fiji/ImageJ and merged into a composite. The area of interest is isolated, brightness and contrast adjusted and individual channels are toggled on and off to determine colocalization with the cell counter tool. Total numbers of neurons in both channels are counted over multiple slices in the A-P axis using the cell counter tool and the number of neurons colocalized is reported as a percent of total eGFP neurons.

### Surgeries for functional validation of connectivity

*TRN*: PV-Cre mice between 10–16 weeks were anesthetized and placed in a stereotaxic frame. The skull was leveled at bregma and lambda and a mounted drill was used to create holes in the skull over the TRN and dorsal striatum based off bregma. A Hamilton syringe (33 gauge needle) was slowly lowered to the TRN (AP −0.58, ML +−1.25, DV −3.5) and 0.1 µl of AAV9-Ef1a-DIO-ChR2 (H134R)-eYFP (titer ~2×10^12^, Penn Vector Core; RRID: SCR_015406) was injected over 10 min. The needle was left in place for 5 min. With a different syringe 1 µl of AAV9-FLEX-tdTomato (titer ~2×10^12^, UNC Vector Core; RRID: SCR_002448) was injected into the dorsal striatum (AP +0.5, ML +−1.9, DV-2.25) to label PV interneurons in a PV-Cre mouse. The AAV9-FLEX-tdTomato was never observed in TRN. In an additional approach to validate TRN connectivity 0.5 µl of AAVretro-EF1a-Flp (UNC Vector Core; RRID: SCR_002448) is injected in dorsal striatum (AP + 0.5, ML +−1.9, DV-2.25) and during the same surgery 0.1 µl of AAV9-hsyn-Con-Fon-hChR2-eYFP (UNC Vector Core; RRID: SCR_002448) is injected in TRN (AP −0.58, ML +−1.25, DV −3.5). Additionally, Sst-IRES-Cre (*Sst*) (Jackson stock # 028864, RRID:IMSR_JAX:028864) mice were injected with AAV9-FLEX-eGFP in the TRN like previous. *GPe:* For GPe to ChAT and PV interneuron recordings 0.2 µl of AAV9-hsyn-ChR2-eYFP (Penn Vector Core; RRID: SCR_015406) was injected in the GPe (AP −0.46, ML +−1.9, DV −3.3) similar to above. 1 µl of AAV9-FLEX-tdTomato (titer ~2×10^12^, UNC Vector Core; RRID: SCR_002448) was injected into the dorsal striatum (AP +0.5, ML +−1.8, DV-2.25) of a ChAT-Cre or PV-Cre mouse to identify ChAT and PV interneurons, respectively. *PPN*: For validation of PPN to striatal interneuron connectivity we injected a small volume (0.1 µl) of AAV9-hsyn-ChR2-eYFP in PPN (AP −4.48, ML +−1.1, DV −3.2) (Penn Vector Core; RRID: SCR_015406) and then injected 1 µl of AAV9-FLEX-tdTomato (AP +0.5, ML +−1.9, DV −2.25) (UNC Vector Core; RRID: SCR_002448) in the striatum in a ChAT-Cre or PV-Cre animal to identify ChAT and PV interneurons.

### Ex vivo brain slice electrophysiology

Two weeks following AAV injection, mice are anesthetized with ketamine/xylazine and transcardially perfused with ice cold, bubbling (95% O_2_/5% CO_2_) NMDG cutting solution [consisting of (in mM): NMDG 105, HCl 105, KCl 2.5, NaH_2_PO_4_ 1.2, NaHCO_3_ 26, Glucose 25, Sodium L-Ascorbate 5, Sodium Pyruvate 3, Thiourea 2, MgSO_4_ 10, CaCl_2_ 0.5, 300mOsm, pH = 7.4]. The brain is blocked coronally or sagittally with a brain matrix (Zivic Instruments; Pittsburg, PA) and acute coronal/sagittal slices (300 µm) were cut on a vibratome (VT1000S, Leica Microsystems; Buffalo Grove, IL) through the striatum in ice cold, bubbling NMDG based cutting solution. Slices were allowed to recover for 15 min at 32°C in bubbling NMDG cutting solution. Slices were then transferred to a holding chamber consisting of normal ACSF [consisting of (in mM): NaCl 125, KCl 2.5, NaH_2_PO_4_ 1.25, NaHCO_3_ 25, D-Glucose 12.5, MgCl_2_ 1, CaCl_2_ 2, pH = 7.4, 295 mOsm] at 28°C. After at least one hour of recovery the slices were placed in a chamber and aCSF was perfused over the slices at ~2 mL/min. PV (+) cells were visualized under IR- DIC optics (Ziess Axioskop2; Oberkocken, Germany) at 40x and confirmed to express tdTomato with brief confirmation in the epifluorescent channel. In some experiments neighboring PV (-) neurons (putative SPNs) were patched. Voltage clamp (28°C) or current clamp (33°C) recordings were made from PV-tdTomato (+) interneurons in the dorsal striatum (~+0.5 mm bregma). 3–4 MΩ patch pipettes (WPI; Sarasota, FL) were pulled from borosilicate glass on a P-97 pipette puller (Sutter Instruments; Novato, CA) and filled with internal solution consisting of (in mM): KMeSO_4_ 135, KCl 5, CaCl_2_ 0.5, HEPES 5, EGTA 5, Mg-ATP 2, Na-GTP 0.3, pH = 7.3, 305mOsm) for current clamp studies. For PV voltage clamp studies to examining TRN connectivity IPSCs were isolated with a high Cl- based internal solution consisting of (in mM): CsCl 120, CsMeSO_3_ 15, NaCl 8, HEPES 10, EGTA 0.5, QX-314 5, Mg-ATP 2, Na-GTP 0.3, pH 7.3, 305mOsm. Following five minutes post break in paired light pulses (473 nm, 5–25 mW/mm^2^, 2.5 ms, 50 ms ISI) were delivered through a 200 µm glass fiber optic (Thor Labs; Newton, NJ) positioned close to the recorded cell (50–150 µm) at 0.05 Hz using a 473 nm blue DPSS laser system (Laserglow Technologies, Toronto, ON). Twenty sweeps (0.05 Hz) were collected to determine latency and CV. The cell was held at −70 mV and light evoked currents are collected after bath application of 10 µM NBQX and 50 µM DL-APV (MilliporeSigma, St. Louis, MO) to block AMPAR and NMDAR-mediated transmission, respectively. IPSCs were collected 8–10 min following the wash in of drugs. 50 µM-100µM picrotoxin (MilliporeSigma, St. Louis, MO) was added to the bath to block fast GABA_A_R transmission and confirm IPSC. Series resistance was initially compensated and monitored continuously throughout the experiment and the data were rejected if the parameters changed by more than 20% over the duration of the recording.

For ChAT, PV and SPN IPSC voltage clamp studies Cs+ methanesulfonate internal solution consisting of (in mM): CsMeSO3 (120), NaCl (5), TEA-Cl (10), HEPES (10), QX-314 (5) EGTA (1.1), Mg-ATP (4), Na-GTP (0.3), pH = 7.2–7.3, 305mOsm paired with holding the cell at −10 mV. Light evoked (473 nm, 5–25 mW/mm^2^, 2.5 ms, 50 ms ISI) IPSCs are collected in the presence of 10 µM NBQX and 50 µM DL-APV to block AMPAR and NMDAR-mediated transmission, respectively. 50 µM-100µM picrotoxin was added to the bath to block fast GABA_A_R transmission and confirm IPSCs. Pairs of ChAT-Cre tdTomato positive or PV-Cre tdTomato positive and neighboring tdTomato negative putative SPNs (<100 µm) are recorded from the same slice. For PPN connectivity experiments Cs + methanesulfonate internal is used like above. Pairs of ChAT and SPN (<100 um) are used for connectivity probability. A cell is considered connected if it has a visible, reliable current (20 sweeps, 0.05 Hz) with onset latency less than 6 ms post laser on. Excitatory and inhibitory currents are determined following wash on of AMPAR/NMDAR antagonists and GABA_A_R antagonists, respectively. Voltage-clamp recordings were performed using a Multiclamp 700A (Molecular Devices; Sunnyvale, CA), digitized (Digidata 1440; Molecular Devices; Sunnyvale, CA) at 10 kHz and filtered at 2 kHz.

For GPe current clamp studies a potassium methane sulfonate internal solution is used. To avoid run down of spiking in ChAT interneuron in whole cell current clamp mode recordings were collected in the first 15 min following break in or in loose patch configuration. Three second threshold current injections (+150–350 pA) were given to drive the PV neuron to fire consistent APs. On interleaved trials 1 s constant or 20 Hz blue light stimulation (473 nm, 5–10 mW/mm^2^) was given to activate GPe ChR2 (+) fibers in the striatum and spiking inhibition was quantified over many sweeps. Perievent time histograms (PETH) are constructed, aligned to laser on and smoothed in MATLAB (Natick, MA). Average spiking during one second stimulation or control (no laser) are compared. Rebound window (1.8–2.9 s average following laser on) is used for rebound statistics. Current clamp recordings were filtered and digitized at 10 kHz and collected with pClamp 9 (Molecular Devices; Sunnyvale, CA; RRID: SCR_011323). Data was analyzed with Clampfit nine and custom MATLAB (RRID: SCR_001622) scripts (see source code MATLAB file).

### In vivo electrophysiology

Striatal neurons were recorded as previously described (Jin et al., 2014). Briefly, mice were lightly anesthetized using isoflourane (4% induction; 1–2% sustained) and were placed in a stereotactic frame. For electrophysiological recording, we utilized electrode arrays (Innovative Neurophysiology Inc.; Durham, NC) of 16 tungsten contacts (2 × 8) that were 35 µm in diameter. Electrodes were spaced 150 µm apart in the same row and 200 µm apart between two rows. Total length of electrodes was 5 mm. Each array with an optic fiber directly attached was employed. The tip of the fiber was ~200 µm away from the tips of the electrodes and the optic fiber was firmly fixed to the array for the duration of each recording session. Array targeting dorsal striatum (+0.5 AP,±1.5 ML, - 2.0 ~ 2.2 DV) was incrementally lowered into dorsal striatum. Silver grounding wire was attached to skull screws.

Neural activity was recorded using the MAP system (Plexon Inc.; Dallas, TX). The spike activities were initially online sorted with a built-in algorithm (Plexon Inc., Dallas, TX). Only spikes with stereotypical waveforms clearly distinguished from noise and with relatively high signal-to-noise ratio were tagged and saved for further analysis. After the recording session, the recorded spikes were further isolated into individual units by an offline sorting software (Offline Sorter, Plexon Inc, Dallas, TX). Each individual unit displayed a clear refractory period in the inter-spike interval histogram, with no spikes during the refractory period (larger than 1.3 ms).

To optogenetically stimulate GPe or TRN terminals within striatum, we injected non-floxed version of AAV-ChR2 virus (0.2 µl of AAV9-hsyn-ChR2-eYFP, Penn Vector Core) into the GPe (AP −0.46, ML + −1.9, DV −3.3) or a Cre-dependent AAV-ChR2 in a PV-Cre mouse for TRN (AP −0.58, ML + −1.25, DV −3.5) (0.1 µl of AAV9-Ef1a-DIO-ChR2(H134R)-eYFP, Penn Vector Core) (titer ~2×10^12^). For each recording session, blue laser stimulation was delivered through the optic fiber from a 473 nm laser (Laserglow Technologies, Toronto, ON) via a fiber-optic patch cord, and the neuronal responses were simultaneously recorded. The stimulation patterns included 1 s constant light and 20 or 50 Hz (10 ms pulse width, 20 or 50 pulses in 1 s). The inter-stimulation interval was 4 s and each stimulation pattern was repeated for 30 trials. The laser power was adjusted carefully (~3.0–5.0 mW) to drive reliable response.

### Statistics

Statistics were conducted in Graph Pad Prism 6.01 (La Jolla, CA; RRID: SCR_002798). Student two-tailed t-test or non-parametric Mann Whitney U Test was conducted when distributions significantly deviated from normal distributions. Two-way ANOVA with Sidak’s multiple comparisons correction was used to analyze whole brain input regions and cortical layer distributions.

## Additional files

Source code 1. MATLAB script for perievent time histogram (PETH) plot. Plot PETH of ChAT or PV interneuron firing rates aligned to laser on (time zero).

Transparent reporting form

### Data availability

All data generated or analyzed during this study are included in the manuscript and supporting files.

## References

[bib1] Adler A, Katabi S, Finkes I, Prut Y, Bergman H (2013). Different correlation patterns of cholinergic and GABAergic interneurons with striatal projection neurons. Frontiers in Systems Neuroscience.

[bib2] Aoki S, Liu AW, Zucca A, Zucca S, Wickens JR (2015). Role of striatal cholinergic interneurons in set-shifting in the rat. Journal of Neuroscience.

[bib3] Aosaki T, Tsubokawa H, Ishida A, Watanabe K, Graybiel AM, Kimura M (1994). Responses of tonically active neurons in the primate's striatum undergo systematic changes during behavioral sensorimotor conditioning. The Journal of Neuroscience.

[bib4] Apicella P (2007). Leading tonically active neurons of the striatum from reward detection to context recognition. Trends in Neurosciences.

[bib5] Arias-García MA, Tapia D, Laville JA, Calderón VM, Ramiro-Cortés Y, Bargas J, Galarraga E (2017). Functional comparison of corticostriatal and thalamostriatal postsynaptic responses in striatal neurons of the mouse. Brain Structure and Function.

[bib6] Assous M, Kaminer J, Shah F, Garg A, Koós T, Tepper JM (2017). Differential processing of thalamic information via distinct striatal interneuron circuits. Nature Communications.

[bib7] Berke JD (2011). Functional properties of striatal fast-spiking interneurons. Frontiers in Systems Neuroscience.

[bib8] Bevan MD, Booth PA, Eaton SA, Bolam JP (1998). Selective innervation of neostriatal interneurons by a subclass of neuron in the globus pallidus of the rat. The Journal of Neuroscience.

[bib9] Bradfield LA, Bertran-Gonzalez J, Chieng B, Balleine BW (2013). The thalamostriatal pathway and cholinergic control of goal-directed action: interlacing new with existing learning in the striatum. Neuron.

[bib10] Brown HD, Baker PM, Ragozzino ME (2010). The parafascicular thalamic nucleus concomitantly influences behavioral flexibility and dorsomedial striatal acetylcholine output in rats. Journal of Neuroscience.

[bib11] Burguière E, Monteiro P, Mallet L, Feng G, Graybiel AM (2015). Striatal circuits, habits, and implications for obsessive-compulsive disorder. Current Opinion in Neurobiology.

[bib12] Chang HT, Kita H (1992). Interneurons in the rat striatum: relationships between parvalbumin neurons and cholinergic neurons. Brain Research.

[bib13] Clemente-Perez A, Makinson SR, Higashikubo B, Brovarney S, Cho FS, Urry A, Holden SS, Wimer M, Dávid C, Fenno LE, Acsády L, Deisseroth K, Paz JT (2017). Distinct thalamic reticular cell types differentially modulate normal and pathological cortical rhythms. Cell Reports.

[bib14] Cowen SL, Davis GA, Nitz DA (2012). Anterior cingulate neurons in the rat map anticipated effort and reward to their associated action sequences. Journal of Neurophysiology.

[bib15] Dautan D, Huerta-Ocampo I, Witten IB, Deisseroth K, Bolam JP, Gerdjikov T, Mena-Segovia J (2014). A major external source of cholinergic innervation of the striatum and nucleus accumbens originates in the brainstem. Journal of Neuroscience.

[bib16] Ding J, Peterson JD, Surmeier DJ (2008). Corticostriatal and thalamostriatal synapses have distinctive properties. Journal of Neuroscience.

[bib17] Ding JB, Guzman JN, Peterson JD, Goldberg JA, Surmeier DJ (2010). Thalamic gating of corticostriatal signaling by cholinergic interneurons. Neuron.

[bib18] English DF, Ibanez-Sandoval O, Stark E, Tecuapetla F, Buzsáki G, Deisseroth K, Tepper JM, Koos T (2012). GABAergic circuits mediate the reinforcement-related signals of striatal cholinergic interneurons. Nature Neuroscience.

[bib19] Freund TF, Buzsáki G (1996). Interneurons of the hippocampus. Hippocampus.

[bib20] Gage GJ, Stoetzner CR, Wiltschko AB, Berke JD (2010). Selective activation of striatal fast-spiking interneurons during choice execution. Neuron.

[bib21] Garas FN, Shah RS, Kormann E, Doig NM, Vinciati F, Nakamura KC, Dorst MC, Smith Y, Magill PJ, Sharott A (2016). Secretagogin expression delineates functionally-specialized populations of striatal parvalbumin-containing interneurons. eLife.

[bib22] Gerfen CR, Engber TM, Mahan LC, Susel Z, Chase TN, Monsma FJ, Sibley DR (1990). D1 and D2 dopamine receptor-regulated gene expression of striatonigral and striatopallidal neurons. Science.

[bib23] Gittis AH, Berke JD, Bevan MD, Chan CS, Mallet N, Morrow MM, Schmidt R (2014). New roles for the external globus pallidus in basal ganglia circuits and behavior. Journal of Neuroscience.

[bib24] Gittis AH, Nelson AB, Thwin MT, Palop JJ, Kreitzer AC (2010). Distinct roles of GABAergic interneurons in the regulation of striatal output pathways. Journal of Neuroscience.

[bib25] Glajch KE, Kelver DA, Hegeman DJ, Cui Q, Xenias HS, Augustine EC, Hernández VM, Verma N, Huang TY, Luo M, Justice NJ, Chan CS (2016). Npas1+ pallidal neurons target striatal projection neurons. Journal of Neuroscience.

[bib26] Goldberg JA, Ding JB, Surmeier DJ (2012). Muscarinic modulation of striatal function and circuitry. Handbook of Experimental Pharmacology.

[bib27] Gonzales KK, Pare JF, Wichmann T, Smith Y (2013). GABAergic inputs from direct and indirect striatal projection neurons onto cholinergic interneurons in the primate putamen. Journal of Comparative Neurology.

[bib28] Gourley SL, Olevska A, Zimmermann KS, Ressler KJ, Dileone RJ, Taylor JR (2013). The orbitofrontal cortex regulates outcome-based decision-making via the lateral striatum. European Journal of Neuroscience.

[bib29] Graybiel AM (2000). The basal ganglia. Current Biology.

[bib30] Gremel CM, Costa RM (2013). Orbitofrontal and striatal circuits dynamically encode the shift between goal-directed and habitual actions. Nature Communications.

[bib31] Guo Q, Wang D, He X, Feng Q, Lin R, Xu F, Fu L, Luo M (2015). Whole-brain mapping of inputs to projection neurons and cholinergic interneurons in the dorsal striatum. Plos One.

[bib32] Hadland KA, Rushworth MF, Gaffan D, Passingham RE (2003). The anterior cingulate and reward-guided selection of actions. Journal of Neurophysiology.

[bib33] Halassa MM, Acsády L (2016). Thalamic inhibition: diverse sources, diverse scales. Trends in Neurosciences.

[bib34] Hayden BY, Platt ML (2010). Neurons in anterior cingulate cortex multiplex information about reward and action. Journal of Neuroscience.

[bib35] Hernández VM, Hegeman DJ, Cui Q, Kelver DA, Fiske MP, Glajch KE, Pitt JE, Huang TY, Justice NJ, Chan CS (2015). Parvalbumin+ neurons and Npas1+ neurons are distinct neuron classes in the mouse external globus pallidus. Journal of Neuroscience.

[bib36] Hikosaka O, Nakahara H, Rand MK, Sakai K, Lu X, Nakamura K, Miyachi S, Doya K (1999). Parallel neural networks for learning sequential procedures. Trends in Neurosciences.

[bib37] Hintiryan H, Foster NN, Bowman I, Bay M, Song MY, Gou L, Yamashita S, Bienkowski MS, Zingg B, Zhu M, Yang XW, Shih JC, Toga AW, Dong HW (2016). The mouse cortico-striatal projectome. Nature Neuroscience.

[bib38] Howard CD, Li H, Geddes CE, Jin X (2017). Dynamic nigrostriatal dopamine biases action selection. Neuron.

[bib39] Jin X, Costa RM (2015). Shaping action sequences in basal ganglia circuits. Current Opinion in Neurobiology.

[bib40] Jin X, Tecuapetla F, Costa RM (2014). Basal ganglia subcircuits distinctively encode the parsing and concatenation of action sequences. Nature Neuroscience.

[bib41] Johnston K, Levin HM, Koval MJ, Everling S (2007). Top-down control-signal dynamics in anterior cingulate and prefrontal cortex neurons following task switching. Neuron.

[bib42] Kalanithi PS, Zheng W, Kataoka Y, DiFiglia M, Grantz H, Saper CB, Schwartz ML, Leckman JF, Vaccarino FM (2005). Altered parvalbumin-positive neuron distribution in basal ganglia of individuals with Tourette syndrome. PNAS.

[bib43] Kepecs A, Fishell G (2014). Interneuron cell types are fit to function. Nature.

[bib44] Kimura M, Minamimoto T, Matsumoto N, Hori Y (2004). Monitoring and switching of cortico-basal ganglia loop functions by the thalamo-striatal system. Neuroscience Research.

[bib45] Kimura M, Rajkowski J, Evarts E (1984). Tonically discharging putamen neurons exhibit set-dependent responses. PNAS.

[bib46] Koós T, Tepper JM (1999). Inhibitory control of neostriatal projection neurons by GABAergic interneurons. Nature Neuroscience.

[bib47] Lapper SR, Bolam JP (1992). Input from the frontal cortex and the parafascicular nucleus to cholinergic interneurons in the dorsal striatum of the rat. Neuroscience.

[bib48] Lim SA, Kang UJ, McGehee DS (2014). Striatal cholinergic interneuron regulation and circuit effects. Frontiers in Synaptic Neuroscience.

[bib49] Mallet N, Le Moine C, Charpier S, Gonon F (2005). Feedforward inhibition of projection neurons by fast-spiking GABA interneurons in the rat striatum in vivo. Journal of Neuroscience.

[bib50] Mallet N, Micklem BR, Henny P, Brown MT, Williams C, Bolam JP, Nakamura KC, Magill PJ (2012). Dichotomous organization of the external globus pallidus. Neuron.

[bib51] Mallet N, Schmidt R, Leventhal D, Chen F, Amer N, Boraud T, Berke JD (2016). Arkypallidal cells send a stop signal to striatum. Neuron.

[bib52] Martinez-Gonzalez C, Bolam JP, Mena-Segovia J (2011). Topographical organization of the pedunculopontine nucleus. Frontiers in Neuroanatomy.

[bib53] Mastro KJ, Bouchard RS, Holt HA, Gittis AH (2014). Transgenic mouse lines subdivide external segment of the globus pallidus (GPe) neurons and reveal distinct GPe output pathways. Journal of Neuroscience.

[bib54] Matamales M, Skrbis Z, Hatch RJ, Balleine BW, Götz J, Bertran-Gonzalez J (2016). Aging-related dysfunction of striatal cholinergic interneurons produces conflict in action selection. Neuron.

[bib55] McAlonan K, Brown VJ (2003). Orbital prefrontal cortex mediates reversal learning and not attentional set shifting in the rat. Behavioural Brain Research.

[bib56] Mena-Segovia J, Micklem BR, Nair-Roberts RG, Ungless MA, Bolam JP (2009). GABAergic neuron distribution in the pedunculopontine nucleus defines functional subterritories. The Journal of Comparative Neurology.

[bib57] Minamimoto T, Kimura M (2002). Participation of the thalamic CM-Pf complex in attentional orienting. Journal of Neurophysiology.

[bib58] Nelson AB, Hammack N, Yang CF, Shah NM, Seal RP, Kreitzer AC (2014). Striatal cholinergic interneurons Drive GABA release from dopamine terminals. Neuron.

[bib59] Okada K, Nishizawa K, Fukabori R, Kai N, Shiota A, Ueda M, Tsutsui Y, Sakata S, Matsushita N, Kobayashi K (2014). Enhanced flexibility of place discrimination learning by targeting striatal cholinergic interneurons. Nature Communications.

[bib60] Parnaudeau S, O'Neill PK, Bolkan SS, Ward RD, Abbas AI, Roth BL, Balsam PD, Gordon JA, Kellendonk C (2013). Inhibition of mediodorsal thalamus disrupts thalamofrontal connectivity and cognition. Neuron.

[bib61] Parthasarathy HB, Graybiel AM (1997). Cortically driven immediate-early gene expression reflects modular influence of sensorimotor cortex on identified striatal neurons in the squirrel monkey. The Journal of Neuroscience.

[bib62] Paus T (2001). Primate anterior cingulate cortex: Where motor control, drive and cognition interface. Nature Reviews. Neuroscience.

[bib63] Pinault D (2004). The thalamic reticular nucleus: structure, function and concept. Brain Research Reviews.

[bib64] Prado VF, Janickova H, Al-Onaizi MA, Prado MA (2017). Cholinergic circuits in cognitive flexibility. Neuroscience.

[bib65] Ramanathan S, Hanley JJ, Deniau JM, Bolam JP (2002). Synaptic convergence of motor and somatosensory cortical afferents onto GABAergic interneurons in the rat striatum. The Journal of Neuroscience.

[bib66] Ravel S, Legallet E, Apicella P (1999). Tonically active neurons in the monkey striatum do not preferentially respond to appetitive stimuli. Experimental Brain Research.

[bib67] Saunders A, Huang KW, Sabatini BL (2016). Globus pallidus externus neurons expressing parvalbumin interconnect the subthalamic nucleus and striatal interneurons. Plos One.

[bib68] Scarr E, Gibbons AS, Neo J, Udawela M, Dean B (2013). Cholinergic connectivity: it's implications for psychiatric disorders. Frontiers in Cellular Neuroscience.

[bib69] Schulz JM, Reynolds JN (2013). Pause and rebound: sensory control of cholinergic signaling in the striatum. Trends in Neurosciences.

[bib70] Sciamanna G, Ponterio G, Mandolesi G, Bonsi P, Pisani A (2015). Optogenetic stimulation reveals distinct modulatory properties of thalamostriatal vs corticostriatal glutamatergic inputs to fast-spiking interneurons. Scientific Reports.

[bib71] Shidara M, Richmond BJ (2002). Anterior cingulate: single neuronal signals related to degree of reward expectancy. Science.

[bib72] Sidibé M, Smith Y (1999). Thalamic inputs to striatal interneurons in monkeys: synaptic organization and co-localization of calcium binding proteins. Neuroscience.

[bib73] Silberberg G, Bolam JP (2015). Local and afferent synaptic pathways in the striatal microcircuitry. Current Opinion in Neurobiology.

[bib74] Smith JB, Klug JR, Ross DL, Howard CD, Hollon NG, Ko VI, Hoffman H, Callaway EM, Gerfen CR, Jin X (2016). Genetic-Based dissection unveils the inputs and outputs of striatal patch and matrix compartments. Neuron.

[bib75] Smith Y, Raju DV, Pare JF, Sidibe M (2004). The thalamostriatal system: a highly specific network of the basal ganglia circuitry. Trends in Neurosciences.

[bib76] Stalnaker TA, Berg B, Aujla N, Schoenbaum G (2016). Cholinergic interneurons use orbitofrontal input to track beliefs about current state. Journal of Neuroscience.

[bib77] Straub C, Tritsch NX, Hagan NA, Gu C, Sabatini BL (2014). Multiphasic modulation of cholinergic interneurons by nigrostriatal afferents. Journal of Neuroscience.

[bib78] Sullivan MA, Chen H, Morikawa H (2008). Recurrent inhibitory network among striatal cholinergic interneurons. Journal of Neuroscience.

[bib79] Tepper JM, Tecuapetla F, Koós T, Ibáñez-Sandoval O (2010). Heterogeneity and diversity of striatal GABAergic interneurons. Frontiers in Neuroanatomy.

[bib80] Tervo DG, Hwang BY, Viswanathan S, Gaj T, Lavzin M, Ritola KD, Lindo S, Michael S, Kuleshova E, Ojala D, Huang CC, Gerfen CR, Schiller J, Dudman JT, Hantman AW, Looger LL, Schaffer DV, Karpova AY (2016). A designer AAV variant permits efficient retrograde access to projection neurons. Neuron.

[bib81] Threlfell S, Lalic T, Platt NJ, Jennings KA, Deisseroth K, Cragg SJ (2012). Striatal dopamine release is triggered by synchronized activity in cholinergic interneurons. Neuron.

[bib82] Wall NR, De La Parra M, Callaway EM, Kreitzer AC (2013). Differential innervation of direct- and indirect-pathway striatal projection neurons. Neuron.

[bib83] Wall NR, De La Parra M, Sorokin JM, Taniguchi H, Huang ZJ, Callaway EM (2016). Brain-Wide maps of synaptic input to cortical interneurons. Journal of Neuroscience.

[bib84] Walton ME, Devlin JT, Rushworth MF (2004). Interactions between decision making and performance monitoring within prefrontal cortex. Nature Neuroscience.

[bib85] Wang HL, Morales M (2009). Pedunculopontine and laterodorsal tegmental nuclei contain distinct populations of cholinergic, glutamatergic and GABAergic neurons in the rat. European Journal of Neuroscience.

[bib86] Wickersham IR, Finke S, Conzelmann KK, Callaway EM (2007). Retrograde neuronal tracing with a deletion-mutant rabies virus. Nature Methods.

[bib87] Yin HH, Knowlton BJ (2006). The role of the basal ganglia in habit formation. Nature Reviews Neuroscience.

